# Solution of road network problem with the help of *m*-polar fuzzy graph using isometric and antipodal concept

**DOI:** 10.1038/s41598-023-33071-9

**Published:** 2023-04-20

**Authors:** Uttam Mondal, Tanmoy Mahapatra, Qin Xin, Madhumangal Pal

**Affiliations:** 1 Department of Applied Mathematics with Oceanology and Computer Programming, Vidyasagar University, Midnapore, 721102 India; 2 Department of Mathematics, Ramkrishna Mahato Govt. Engg. College, Purulia, 723103 India; 3 Faculty of Science and Technology, University of the Faroe Islands, Vestara Bryggja 15, Tórshavn, FO-100, Faroe Islands

**Keywords:** Mathematics and computing, Computer science

## Abstract

The isometry in crisp graph theory is a well-known fact. But, isometry under a fuzzy environment was developed recently and studied many facts. In a *m*-polar fuzzy graph, we have to think *m* components for each node and edge. Since, in our consideration, we consider *m* components for each nodes as well as edges, therefore we can not handle this type of situation using fuzzy model as their is a single components for this concept. Again, we can not apply bipolar or intuitionistic fuzzy graph model as each edges or nodes have just two components. Thus, these *m*PFG models give more efficient fuzziness results than other fuzzy model. Also, it is very interesting to develop and analyze such types of *m*PFGs with examples and related theorems. Considering all those things together, we have presented isometry under a *m*-polar fuzzy environment. In this paper, we have discussed the isometric *m*-polar fuzzy graph along with many exciting facts about it. Metric space properties have also been implemented on *m*-polar fuzzy isometric graph. We also have initiated a generalized fuzzy graph, namely antipodal *m*-polar fuzzy graphs, along with several issues. The degree of it is also presented along with edge regularity properties. We also give a relation between *m*-polar fuzzy antipodal graphs and their underlying crisp graphs. Its properties have also been discussed on *m*-polar fuzzy odd as well as even cycles, complete graphs, etc. Finally, a real-life application on a road network system in a *m*-polar fuzzy environment using the $\mu$-distance concept is also presented.

## Introduction

### Research background and related works

In technical development, fuzzy graph (FG) theory has an important role. The way of many rule-based expert systems for engineers have been made from FG theory. It is seen in the maximum time that the graph theory is found as an essential part of connectivity in some fields of geometry, algebra, topology, number theory, computer science, operations research and optimization. In 1975, first Rosenfeld^32^ considered the relations on fuzzy sets, called fuzzy relations and developed the concept of FGs. Many definitions and conception are demonstrated thereafter in Mathew and Sunitha^26^, Anjali and Mathew^8^, Mordeson and Nair^27^, Sunitha and Mathew^35^, mostly under operations on FGs, fuzzy paths, complement of a FG, fuzzy sub-graphs, fuzzy trees. Fuzzy planar graph is initiated by Samanta and Pal^33^ and also Samanta et al. introduced fuzzy coloring of FGs^34^. A new fuzzy graph parameter for the comparison of human trafficking chains is initiated by Arya, Mathew and Mordeson^9^. Krishnaveni and Balasundaram^19^ gave the concept of generating fuzzy graph based multi-document summary of text based learning materials. Many works of graph have been done on soft environment like characterizations of certain types of type 2 soft graph^16^, a new type-2 soft set: type-2 soft graphs and their applications^17^, new group-based generalized interval-valued q-rung orthopair fuzzy soft aggregation operators and their applications in sports decision-making problems^18^ etc.

In our world, many real problems have been solved using data which comes from different origins. This type of data collection represents multi-polarity. This type of polarity can not be structured well by the conception of FGs or bipolar FGs. To reduce the situation, the conception of the *m*-polar fuzzy set is imposed on graphs to elaborate on the different origins. Firstly, *m*-polar fuzzy graph (*m*PFG) was initiated by Chen et al.^10^. After that Ghorai and Pal studied some operations and density of *m*PFGs^12^ and then they respectively introduced the concept of *m*PF planar graphs^14^, faces and dual of *m*PF planar graphs^13^ and some isomorphic properties of *m*PFGs^15^. The genus value of *m*PFGs is initiated by Mandal et al.^25^. Next, several types of arcs were studied by Mandal et al. on *m*PFG^24^. The *m*-polarity of FGs, line graphs and fuzzy labeling graphs were studied by Akram and Adeel^1,4^. Fuzzy hypergraphs and competition graphs in *m*-polar environment are introduced by Akram and Sarwar^5,6^. A few features of edge were discussed by Akram et al. on *m*PFG^2^ and also Akram^3^ discussed many concepts on *m*PFG. Next, Mahapatra and Pal introduced the concepts of fuzzy colouring of *m*PFG^20^, *m*-polar fuzzy tolerance graph^22^ and also they investigated on *m*-polar fuzzy threshold graph and gave an application on resource power controlling system^23^. Clustering algorithm with strength of connectedness for *m*-polar fuzzy network models is studied by Akram et al.^7^.

The conception of isometry in FGs was initiated by Nagoorgani and Malarvizhi, who studied some features of it^11^. The conception of isometry on interval-valued FGs was introduced by Rashmanlou and Pal^30^. Later on, Radha and Indumathi studied regular and isometric FGs^29^. The notion of antipodal FG was initiated by Nagoorgani and Malarvizhi^11^. Rashmanlou and Pal^31^ initiated the conception of antipodal interval-valued FGs and investigated some properties on it. For fundamental terminologies and definition see^28^.

### Motivation and contribution of the work

In our real life, many problems have been solved using data which comes from different origins or sources. This type of data collection represents multi-polarity. In this type of polarity, we can not be structured well by the conception of fuzzy models, intuitionistic fuzzy models or bipolar fuzzy models. For example, if we consider a road networking model which assures minimum travel time of passengers. For this, we assign the node membership value (MV) based on the situation of the roads as (jam on the road, transport availability on the road, condition of the road). In nature, these terms are uncertain. To represent this situation, we need to use the 3-polar fuzzy model. Since, in our consideration, we consider three components for each nodes as well as edges, therefore we can not handle this type of situation using fuzzy model as their is a single components for this concept. Again, we can not apply bipolar or intuitionistic fuzzy graph model as each edges or nodes have just two components. Thus, these *m*PFG models give more efficient fuzziness results than other fuzzy model. Also, it is very interesting to develop and analyze such types of *m*PFGs with examples and related theorems. These definitions and theorems are definitely improving the existing concepts of *m*PFGs and are more reliable for solving any complicated real-life problem.

We have discussed the *m*-polar fuzzy isometric graph along with many exciting facts about it. Metric space properties have also been implemented on *m*-polar fuzzy isometric graph. We also initiate a generalized fuzzy graph, namely antipodal *m*-polar fuzzy graphs, along with several facts. The degree of it is also presented along with edge regularity properties. We also give a relation between *m*-polar fuzzy antipodal graphs and their underlying crisp graphs. The properties have also been discussed on *m*-polar fuzzy odd as well as even cycles, complete graphs, etc. Finally, a real-life application based on a road network system in *m*-polar fuzzy environment using the concept of $\mu$-length is also presented. This *m*PFG models give more efficient fuzziness results than other fuzzy model. Also, it is very interesting to develop and analyze such types of *m*PFGs with examples and related theorems. These definitions and theorems are definitely improving the existing concepts of *m*PFGs and are more reliable for solving any complicated real-life problem.

The formation of this article is as follows: Section 3 mentions some useful concepts which are essential for this article. In Section 4, we have defined the concept of isometric *m*PFG and provided some theorems on their aspect. Also, we have given the distance preserving concept of isometric on *m*PFG. In Section 5, we have introduced a new concept called antipodal *m*PFG; the degree of an edge of an antipodal *m*PFG and edge regularity on antipodal *m*PFG has been studied. We investigated some features based on the above conception. In Section 6, we have discussed an application based on a road networking problem. Some advantages, disadvantages and limitations in the study are given in Section 7. Finally, in Section 8, the conclusion of the study have been presented.

## Preliminaries

Here, we briefly recall again some definitions connected to *m*PFG, like complete *m*PFG, strong *m*PFG, path in *m*PFG, etc.

Throughout this article $p_i:[0,1]^{m}\rightarrow [0,1]$ indicated *i*th material of projection mapping.

### Definition 1

^10^ An *m*PFS *A* (or a $[0,1]^{m}$-set ) on *X* is a mapping $A:X\rightarrow [0,1]^{m}$. *m*(*X*) indicates the set of all *m*-polar fuzzy sets on *X*.

### Definition 2

^10^ Let *A* be an *m*PFS. Then height of *A* is indicated by *h*(*A*) and is defined by

$$\begin{aligned} (\sup _{x\in A}p_1\,{\circ }\, A(x),\sup _{x\in A}p_2\,{\circ }\, A(x),\ldots ,\sup _{x\in A}\,p_m{\circ } \,A(x)) \end{aligned}$$.

### Definition 3

^10^ The support of an *m*PFS *A* is defined as $supp(A)=\{c\in A:p_i\,{\circ }\, B(c)>0\}$, $i=1,2,\ldots ,m$, indicated by *supp*(*A*), where $B:A\rightarrow [0,1]^m$ is a mapping.

Clearly, $supp(A)=\phi$ iff $A=\phi$ and $supp(A)\ne \phi$ iff $A\ne \phi$. Therefore, *A* and *supp*(*A*) is equivalent to *m*PFS.

### Definition 4

^15^ An *m*PFG $\varGamma =({\tilde{A}},\sigma ,\mu )$ having UCG $\varGamma ^*=({\tilde{A}},{\tilde{B}})$, where $\sigma : {\tilde{A}}\rightarrow [0,1]^m$ and $\mu :\widetilde{{\tilde{A}}\times {\tilde{A}}}\rightarrow [0,1]^m$ indicate an *m*PFS of ${\tilde{A}}$ and $\widetilde{{\tilde{A}}\times {\tilde{A}}}$ respectively and which follows the relation such that for all $i=1,2,\dots ,m$, $p_i\,{\circ }\, \mu (b,c)\le \{p_i\,{\circ }\, \sigma (b)\wedge p_i\,{\circ }\, \sigma (c)\}$ for all $(b,c)\in \widetilde{{\tilde{A}}\times {\tilde{A}}}$ as well as $\mu (b,c)=\mathbf{{0}}$ for all $(b,c)\in (\widetilde{{\tilde{A}}\times {\tilde{A}}}-{\tilde{B}})$.

### Definition 5

^12^ The *m*PFG $\varGamma =({\tilde{A}},\sigma ,\mu )$ is conferred as complete *m*PFG provided $p_i\,{\circ }\, \mu (b,c) =\{p_i\,{\circ }\, \sigma (b) \wedge p_i\,{\circ }\, \sigma (c)\},$ for all $b,c \in {\tilde{A}}, ~i=1,2,\dots ,m$.

### Definition 6

^15^ The *m*PFG $\varGamma =({\tilde{A}},\sigma ,\mu )$ is conferred as a *m*PF strong graph if

$$\begin{aligned} p_i\,{\circ }\, \mu (b,c) = \{p_i\,{\circ }\, \sigma (b)\wedge p_i\,{\circ }\, \sigma (c)\}, \end{aligned}$$

for all $$~ (b,c) \in {\tilde{B}}, ~i=1,2,\dots ,m$$.

### Definition 7

^12^ An *m*PFG $G=(V,\sigma ,\mu )$ is said to be bipartite if the vertex set *V* can be partitioned into two non-empty sets $V_1$ and $V_2$ such that for each $i=1,2,\ldots m$, $p_i\,{\circ }\, \mu (a,b)> 0$ if $a,b \in V_1$ or $a,b \in V_2$.

### Definition 8

^12^ Consider $\varGamma =({\tilde{A}},\sigma ,\mu )$ and $\varGamma '=(\tilde{A'},\sigma ',\mu ')$ be two *m*PFGs of the underline graphs $\varGamma ^*=({\tilde{A}},{\tilde{B}})$ and ${\varGamma '}^*=(\tilde{A'},\tilde{B'})$ respectively. An isomorphism between $\varGamma$ and $\varGamma '$ is a bijective mapping $f:{\tilde{A}} \rightarrow \tilde{A'}$ which satisfies

$$\begin{aligned} p_i\,{\circ }\, \sigma (t)=p_i\,{\circ }\, \sigma '(f(t)) \text { and } p_i\,{\circ }\, \mu (t,u)=p_i\,{\circ }\, \mu '(f(t),f(u)) \end{aligned}$$

for all $t, u\in {\tilde{A}}$ and every $i=1,2,\dots ,m$. Then $\varGamma$ is called to be isomorphic with $\varGamma '$.

### Definition 9

^12^ A co-weak isomorphism between $\varGamma$ and $\varGamma '$ is a bijective mapping $f:{\tilde{A}} \rightarrow \tilde{A'}$ which is homomorphism that satisfies

$$\begin{aligned} p_i\,{\circ }\, \mu (t,u)=p_i\,{\circ }\, \mu '(f(t),f(u)) \end{aligned}$$

for all $t, u\in {\tilde{A}}$ and for each $i=1,2,\dots ,m$.

### Definition 10

^2^ Let $\varGamma =({\tilde{A}},\sigma ,\mu )$ be an *m*PFG as well as $P: b_1,b_2,\ldots ,b_k$ be a path in $\varGamma$. *S*(*P*) indicates the strength of *P* which is defined as $S(P)=(\min \limits _{1\le i<j\le k} p_1\,{\circ }\, \mu (b_i,b_j),\min \limits _{1\le i<j\le k}p_2\,{\circ }\, \mu (b_i,b_j),\ldots ,\min \limits _{1\le i<j\le k}p_m\,{\circ }\, \mu (b_i,b_j))=(\mu _1^n(b_i,b_j),\mu _2^n(b_i,b_j),\ldots ,\mu _m^n(b_i,b_j))$.

The strength of connectedness of the path in between $b_1$ and $b_k$ is given in the following way: $CONN_\varGamma (b_1,b_k)=(p_1\,{\circ }\,\mu (b_i,b_j)^\infty , p_2\,{\circ }\,\mu (b_i,b_j)^\infty ,\ldots , p_m\,{\circ }\,\mu (b_i,b_j)^\infty )$, where $(p_i\,{\circ }\,\mu (b_i,b_j)^\infty )=\max \limits _{n\in N} (\mu _i^n(b_i,b_j)).$

### Definition 11

^20^ For an *m*PFG $\varGamma =({\tilde{A}},\sigma ,\mu ),$ an edge $(b,c),b,c\in {\tilde{A}}$ is entitled as independently strong if $\frac{1}{2}\{p_i\,{\circ }\,\sigma (b) \wedge p_i\,{\circ }\, \sigma (c)\}\le p_i\,{\circ }\,\mu (b,c),i=1,2,\dots ,m.$ Otherwise, it is said to be an independently weak edge. The strength of the edge (*b*, *c*) is defined by

$$\begin{aligned} p_i\,{\circ }\, I(b,c)=\frac{p_i\,{\circ }\, \mu (b,c)}{p_i\,{\circ }\,\sigma (b) \wedge p_i\,{\circ }\, \sigma (c)}, ~~i=1,2,\dots ,m. \end{aligned}$$

## Isometric concept on *m*-polar fuzzy graphs

A new conception called isometric on *m*PFGs is introduced, in this portion. Isometric means distance preserving in the ordinary sense but under *m*-polar fuzzy environment how it works; we have investigated along with its many interesting ideas.

**Figure 1:**
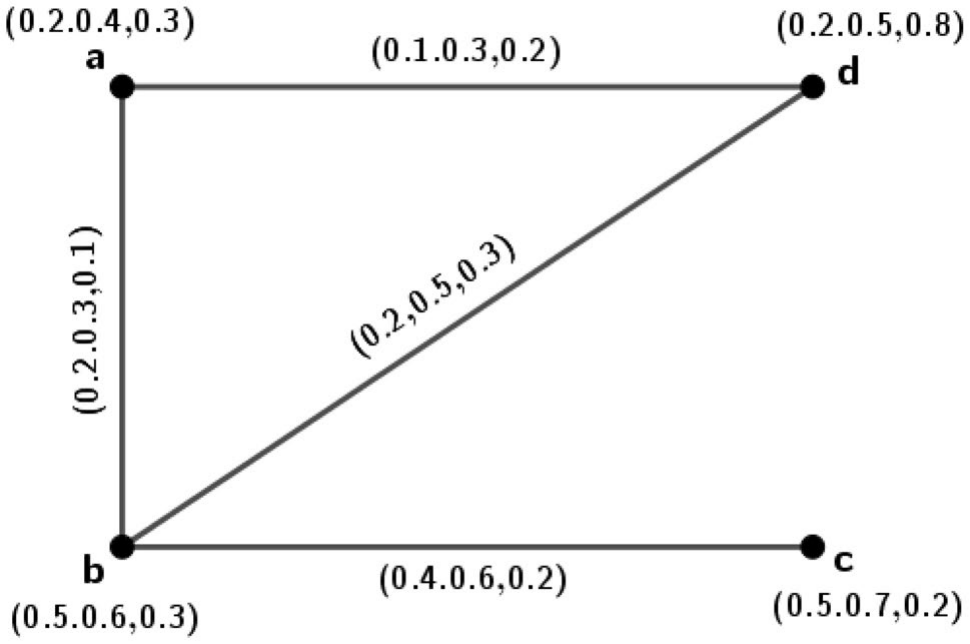
*m*PFG $$\varGamma _1=({\tilde{A}}_1,\sigma _1,\mu _1)$$.

### Definition 12

Let $\varGamma _1=({\tilde{A}}_1,\sigma _1,\mu _1)$ and $\varGamma _2=({\tilde{A}}_2,\sigma _2,\mu _2)$ be two *m*PFGs. $\varGamma _2$ is called to be isometric to $\varGamma _1$ if for each $y\in {\tilde{A}}_1$ if there exists a bijection $\phi _y : {\tilde{A}}_1\rightarrow {\tilde{A}}_2$ such that

$$\begin{aligned} p_i\,{\circ }\,\delta _1(x,y)=p_i\,{\circ }\,\delta _2(\phi _y(x),\phi _y(y)), \end{aligned}$$

$i=1,2,\dots ,m$, for each $x\in {\tilde{A}}_1$ and $\phi _y(x), \phi _y(y)\in {\tilde{A}}_2$.

Here $p_i\,{\circ }\,\delta (x,y)$ is the $\mu$-distance of the path $\rho : x=x_0, x_1, x_2,\ldots , x_{n-1}, x_n=y$, defined by $p_i\,{\circ }\,\delta (x,y)=\wedge \sum _{j=1}^{n}\frac{1}{p_i\,{\circ }\,\mu (x_{j-1},x_j)}$, for each $i=1,2,\dots ,m$.

### Example 1

Let $\varGamma _1=({\tilde{A}}_1,\sigma _1,\mu _1)$ be a 3PFG shown in Fig. 1.

The set of nodes ${\tilde{A}}_1=\{a, b, c, d\}$ such that $\sigma _1(a)=(0.2,0.4,0.3)$, $\sigma _1(b)=(0.5,0.6,0.3)$, $\sigma _1(c)=(0.5,0.7,0.2)$, $\sigma _1(d)=(0.2,0.5,0.8)$ with $\mu _1(a,b)=(0.2,0.3,0.1)$, $\mu _1(b,c)=(0.4,0.6,0.2)$, $\mu _1(a,d)=(0.1,0.3,0.2)$, $\mu _1(b,d)=(0.2,0.5,0.3)$. In this *m*PFG, $\mu$-length for the path $\rho _1: a-b$ is $l(\rho _1)=(\frac{1}{0.2},\frac{1}{0.3},\frac{1}{0.1})=(5,3.3,10)$, $\mu$-length for the path $\rho _2: a-d-b$ is $l(\rho _2)= \big (\frac{1}{0.1}+\frac{1}{0.2},\frac{1}{0.3}+\frac{1}{0.5},\frac{1}{0.2}+\frac{1}{0.3} \big )=(15,5.3,8.3)$. Therefore, $\delta _1(a,b)=(5,3.3,8.3)$.

Similarly, $$\delta _1(a,c)=(7.5,4.7,13.3)$$, $$\delta _1(a,d)=(10,3.3,5)$$, $$\delta _1(b,c)=(2.5,1.6,5)$$, $$\delta _1(b,d)=(5,2,3.3)$$, $$\delta _1(c,d)=(7.5,3.6,8.3)$$.

Let $\varGamma _2=({\tilde{A}}_2,\sigma _2,\mu _2)$ be a 3PFG on the set ${\tilde{A}}_2=\{u, v, w, x\}$ such that $\sigma _2(u)=(0.4,0.6,0.5)$, $\sigma _2(v)=(0.4,0.7,0.4)$, $\sigma _2(w)=(0.6,0.8,0.2)$, $\sigma _2(x)=(0.5,0.7,0.3)$ with $\mu _2(u,v)=(0.2,0.3,0.12)$, $\mu _2(u,x)=(0.1,0.28,0.2)$, $\mu _2(v,x)=(0.2,0.5,0.3)$, $\mu _2(v,w)=(0.4,0.62,0.2)$, $\mu _2(w,x)=(0.13,0.27,0.12)$. We define $\phi : {\tilde{A}}_1\rightarrow {\tilde{A}}_2$ as $\phi (a)=u$, $\phi (b)=v$, $\phi (c)=w$, $\phi (d)=x$. Now, we have $\delta _2(\phi (a),\phi (b))=\delta _2(u,v)=(5,3.3,8.3)=\delta _1(a,b)$. Similarly, $\delta _1(a,c)=\delta _2(\phi (a),\phi (c))$, $\delta _1(a,d)=\delta _2(\phi (a),\phi (d))$, $\delta _1(b,c)=\delta _2(\phi (b),\phi (c))$, $\delta _1(b,d)=\delta _2(\phi (b),\phi (d))$ and $\delta _1(c,d)=\delta _2(\phi (c),\phi (d))$. So, $\phi$ is one-one and onto function that preserves the distance between every pair of vertices in $\varGamma _1$ and $\varGamma _2$. Hence $\varGamma _2$ is isometric to $\varGamma _1$.

### Theorem 1

Isometry on *m*PFG is an equivalence relation.

### Proof

Let $\varGamma _1=({\tilde{A}}_1,\sigma _1,\mu _1)$, $\varGamma _2=({\tilde{A}}_2,\sigma _2,\mu _2)$ and $\varGamma _3=({\tilde{A}}_3,\sigma _3,\mu _3)$ be three *m*PFGs.

Taking a map $I: {\tilde{A}}_1\rightarrow {\tilde{A}}_1$, called identity map, so $\varGamma _1$ is isometric to $\varGamma _1$. Therefore, the relation isometry is reflexive.

For the symmetric relation, let $\varGamma _1$ be isometric to $\varGamma _2$. Then, each $v\in {\tilde{A}}_1$, there exists a bijection $f_v: {\tilde{A}}_1\rightarrow {\tilde{A}}_2$ such that $p_i\,{\circ }\,\delta _1(u,v)=p_i\,{\circ }\,\delta _2(f_v(u),f_v(v))$, for each $i=1,2,\dots ,m$ and for all $u\in {\tilde{A}}_1$. Now, each $f_v(u)$, we have a bijective mapping $f^{-1}_v: {\tilde{A}}_2\rightarrow {\tilde{A}}_1$ such that

$$\begin{aligned} p_i\,{\circ }\,\delta _2(f_v(u),f_v(v))=p_i\,{\circ }\,\delta _1(f^{-1}_v(f_v(u)),f^{-1}_v(f_v(v))), \end{aligned}$$

$i=1,2,\dots ,m$ and for all $f_v(v)\in {\tilde{A}}_1$, i.e.,

$$\begin{aligned} p_i\,{\circ }\,\delta _2(f_v(u),f_v(v))=p_i\,{\circ }\,\delta _1(u,v), \end{aligned}$$

for each $i=1,2,\dots ,m$ and for all $f_v(v)\in {\tilde{A}}_1$. This shows that $\varGamma _2$ is isometric to $\varGamma _1$. Hence, the isometry relation is symmetric.

For the transitivity relation, consider $\varGamma _1$ is isometric to $\varGamma _2$ and $\varGamma _2$ is isometric to $\varGamma _3$. Then each $v\in {\tilde{A}}_1$, there exists a bijection $f_v : {\tilde{A}}_1\rightarrow {\tilde{A}}_2$ such that $p_i\,{\circ }\,\delta _1(v,u)=p_i\,{\circ }\,\delta _2(f_v(v),f_v(u))$, $i=1,2,\dots ,m$ and for all $u\in {\tilde{A}}_1$. Assume that $f_v(v)=v'$. Similarly, each $v'\in {\tilde{A}}_2$, there exists a bijection $g_{v'}: {\tilde{A}}_2\rightarrow {\tilde{A}}_3$ such that $p_i\,{\circ }\,\delta _2(v',u')=p_i\,{\circ }\,\delta _3(g_{v'}(v'),g_{v'}(u'))$, $i=1,2,\dots ,m$ and for all $u'\in {\tilde{A}}_2$. Now if $v\in {\tilde{A}}_1$,

$$\begin{aligned} p_i\,{\circ }\,\delta _1(v,u)&=p_i\,{\circ }\,\delta _2(f_v(v),f_v(u))\\&=p_i\,{\circ }\,\delta _2(v',u')\\&=p_i\,{\circ }\,\delta _3(g_{v'}(v'),g_{v'}(u'))\\&=p_i\,{\circ }\,\delta _3(g_{v'}(f_v(v)),g_{v'}(f_v(u)))\\&=p_i\,{\circ }\,\delta _3(g_{v'}\,{\circ }\, f_v(v),g_{v'}\,{\circ }\, f_v(u)), \end{aligned}$$

$i=1,2,\dots ,m$ and for all $u\in {\tilde{A}}_1$. This shows that $\varGamma _1$ is isometric to $\varGamma _3$. Hence, the isometry relation is transitive. Therefore, the isometry relation on *m*PFGs is an equivalence relation.

### Theorem 2

Let $\varGamma _1$ be isomorphic to $\varGamma _2$. Then $\varGamma _1$ be isometric to $\varGamma _2$.

### Proof

Given $\varGamma _1$ is isomorphic to $\varGamma _2$, then there is a bijection $h: {\tilde{A}}_1\rightarrow {\tilde{A}}_2$ such that $p_i\,{\circ }\, \sigma _1(x)=p_i\,{\circ }\, \sigma _2(h(x))$, $i=1,2,\dots ,m$ and for all $x\in {\tilde{A}}_1$ and $p_i\,{\circ }\, \mu _1(x,y)=p_i\,{\circ }\, \mu _2(h(x),h(y))$, $i=1,2,\dots ,m$ and for all $x, y\in {\tilde{A}}_1$.

Every $u\in {\tilde{A}}_1$, the $\mu$-distance of the path $\rho : u=u_0, u_1, u_2,\ldots , u_{n-1}, u_n=v$ is given by

$$\begin{aligned} p_i\,{\circ }\,\delta _1(u,v)&=\wedge \sum _{j=1}^{n}\frac{1}{p_i\,{\circ }\,\mu _1(u_{j-1},u_j)}\\&=\wedge \sum _{j=1}^{n}\frac{1}{p_i\,{\circ }\,\mu _2(h(u_{j-1}),h(u_j))}\\&=p_i\,{\circ }\,\delta _2(f(u),f(v)), \end{aligned}$$

$i=1,2,\dots ,m$ and for all $v\in {\tilde{A}}_1$. This shows that $\varGamma _1$ is isometric to $\varGamma _2$. Therefore, $\varGamma _1$ is isometric to $\varGamma _2$.

But the converse case of Theorem 2 is not valid, as illustrated in Example 2.

### Example 2

From Example 1, we see that $\varGamma _1$ is isometric to $\varGamma _2$ but $\varGamma _1$ is not isomorphic to $\varGamma _2$. Because, in example 1, $p_i\,{\circ }\, \sigma _1(a)\ne p_i\,{\circ }\,\sigma _2(u)$, for all $a\in {\tilde{A}}_1, u\in {\tilde{A}}_2$, $i=1,2,\dots ,m$ and $p_i\,{\circ }\,\mu _1(a,b)\ne p_i\,{\circ }\,\mu _2(u,v)$, $i=1,2,\dots ,m$, for all $a, b\in {\tilde{A}}_1$ and $u, v\in {\tilde{A}}_2$. So, we can not have any such bijection $h : {\tilde{A}}_1\rightarrow {\tilde{A}}_2$ such that $p_i\,{\circ }\, \sigma _1(x)=p_i\,{\circ }\, \sigma _2(h(x))$, $i=1,2,\dots ,m$ and for all $x\in {\tilde{A}}_1$ and $p_i\,{\circ }\, \mu _1(x,y)=p_i\,{\circ }\, \mu _2(h(x),h(y))$, $i=1,2,\dots ,m$ and for all $x, y\in {\tilde{A}}_1$.

### Proposition 1

If $\varGamma _1$ is co-weak isomorphic to $\varGamma _2$, then $\varGamma _1$ is isometric to $\varGamma _2$.

### Proof

Similar as Theorem 2.

### Theorem 3

Suppose $\varGamma _1=({\tilde{A}}_1,\sigma _1,\mu _1)$ and $\varGamma _2=({\tilde{A}}_2,\sigma _2,\mu _2)$ be two *m*PFGs such that $\varGamma ^*_1$ and $\varGamma ^*_2$ are *k*-regular graphs, $p_i\,{\circ }\, \mu _1$ and $p_i\,{\circ }\, \mu _2$ are constant functions of same constant values $c_i$, for each $i=1,2,\dots ,m$ and $|{\tilde{A}}_1|=|{\tilde{A}}_2|=n$. If $k\ge \frac{n-1}{2}$, then $\varGamma _1$ and $\varGamma _2$ are isometric *m*PFGs.

### Proof

Let us construct an isometric mapping from $\varGamma _1$ to $\varGamma _2$ as follows :

Choose $u\in {\tilde{A}}_1$ and $v\in {\tilde{A}}_2$ arbitrarily. Define $\phi _u: {\tilde{A}}_1\rightarrow {\tilde{A}}_2$ by $p_i\,{\circ }\, \phi _u(u)=v$, for each $i=1,2,\dots ,m$.

Since $\varGamma ^*_1$ and $\varGamma ^*_2$ are *k*-regular, *k* vertices, say, $u_1, u_2, \ldots , u_k$ are at distance 1 from *u* in $\varGamma ^*_1$ and *k* vertices, say, $v_1, v_2,\ldots ,v_k$ are at distance 1 from *v* in $\varGamma ^*_2$.

Next we define $\phi _u(u_j)=v_j$, for each $j=1,2,\dots ,k$.

Since $\varGamma ^*_1$ and $\varGamma ^*_2$ are *k*-regular with $k\ge \frac{n-1}{2}$, $d(\varGamma ^*_1)\le 2$ and $d(\varGamma ^*_2)\le 2$. Therefore, the remaining $(n-1-k)$ vertices, say, $u_{k+1}, u_{k+2}, \ldots , u_{n-1}$ in $\varGamma _1^*$ are at distance 2 from *u* and the remaining $(n-1-k)$ vertices, say, $v_{k+1}, v_{k+2},\ldots , v_{n-1}$ in $\varGamma _2^*$ are at distance 2 from *v*.

Since $p_i\,{\circ }\, \mu _1$ and $p_i\,{\circ }\, \mu _2$ are constant functions of same constant values *c*, using $\mu$-distance,

$$\begin{aligned} p_i\,{\circ }\, \delta _1(u,u_j)&=p_i\,{\circ }\, \mu _1(u,u_j)=c\\ p_i\,{\circ }\, \delta _2(v,v_j)&=p_i\,{\circ }\, \mu _2(v,v_j)=c\\ \text {That is, } p_i\,{\circ }\, \delta _1(u,u_j)&=p_i\,{\circ }\, \delta _2(v,v_j) \end{aligned}$$

for all $$j=1,2,\dots ,k$$.

Since $u_{k+1}, u_{k+2}, \ldots ,u_{n-1}$ are at distance 2 in $\varGamma ^*_1$, we have

$$\begin{aligned} p_i\,{\circ }\, \delta _1(u,u_j)=2c, \end{aligned}$$

for each $i=1,2,\dots ,m$ and $j=k+1,k+2 \ldots , n-1$.

Similarly, $p_i\,{\circ }\, \delta _2(v,v_j)=2c$, for each $i=1,2,\dots ,m$ and for all $j=k+1, k+2,\ldots ,n-1$.

So, $p_i\,{\circ }\, \delta _1(u,u_j)=p_i\,{\circ }\, \delta _2(v,v_j)$, for each $i=1,2,\dots ,m$ and $j=k+1, k+2,\ldots ,n-1$.

Therefore, $p_i\,{\circ }\, \delta _1(u,u_j)=p_i\,{\circ }\, \delta _2(v,v_j)$, for each $i=1,2,\dots ,m$ and $j=k+1, k+2,\ldots ,n-1$.

Since *u* and *v* are arbitrary, $\varGamma _2$ is isometric to $\varGamma _1$. Therefore, $\varGamma _1$ and $\varGamma _2$ are isometric *m*PFGs.

### Theorem 4

Let $\varGamma _1=({\tilde{A}}_1,\sigma _1,\mu _1)$ and $\varGamma _2=({\tilde{A}}_2,\sigma _2,\mu _2)$ be two $(r,r,\ldots ,r)$- regular *m*PFGs such that $p_i\,{\circ }\, \mu _1$ and $p_i\,{\circ }\, \mu _2$ are constant functions of same constant value *c*, for each $i=1,2,\dots ,m$ and $|{\tilde{A}}_1|=|{\tilde{A}}_2|=n$. If $r\ge \frac{n-1}{2}$, then $\varGamma _1$ and $\varGamma _2$ are isometric *m*PFGs.

### Proof

Since $\varGamma _1$ is $(r,r,\ldots ,r)$-regular, therefore, for each $v\in {\tilde{A}}$ and $i=1,2,\dots ,m$

$$\begin{aligned} p_i\,{\circ }\, d_{\varGamma _1}(u)&=r\\ \text {or, } \sum _{u\ne v}p_i\,{\circ }\, \mu _1(u,v)&=r\\ \text {or, } \sum _{u\ne v}c&=r\\ \text {or, } cd_{\varGamma ^*_1}(u)&=r\\ \text {or, } d_{\varGamma ^*_1}(u)&=\frac{r}{c} \end{aligned}$$

Similarly, $d_{\varGamma ^*_1}(u)=\frac{r}{c}$, for all $u\in {\tilde{A}}_2$ and $i=1,2,\dots ,m$. So, $\varGamma ^*_1$ and $\varGamma ^*_2$ are $\frac{r}{c}$-regular. Then proceeding as Theorem 3, $\varGamma _1$ and $\varGamma _2$ are isometric *m*PFGs.

### Theorem 5

Let $\varGamma _1=({\tilde{A}}_1,\sigma _1,\mu _1)$ and $\varGamma _2=({\tilde{A}}_2,\sigma _2,\mu _2)$ be two *m*PFGs such that $p_i\,{\circ }\, \mu _1$ and $p_i\,{\circ }\, \mu _2$ are constant functions of same constant value *c*, for each $i=1,2,\dots ,m$. If $\varGamma _1$ and $\varGamma _2$ are isometric *m*PFGs, then they have the same degree set and same eccentricity set.

### Proof

Since $\varGamma _2$ is isometric to $\varGamma _1$, there exists a bijection $\phi : {\tilde{A}}_1\rightarrow {\tilde{A}}_2$ such that

1 $$\begin{aligned} p_i\,{\circ }\, \delta _1(u,v)=p_i\,{\circ }\, \delta _2(\phi (u),\phi (v)),\nonumber \\ \text { for all } u, v\in {\tilde{A}}_1 \text { and } i=1,2,\dots ,m. \end{aligned}$$

If *u* and *v* are connected by an edge in $\varGamma _1$, then using $\mu$-distance in *m*PFGs,

$$\begin{aligned} p_i\,{\circ }\, \delta _1(u,v)=p_i\,{\circ }\, \mu (u,v)=c, \end{aligned}$$

for each $$i=1,2,\dots ,m$$.

From Eq. (1),

$$\begin{aligned} p_i\,{\circ }\, \delta _2(\phi (u),\phi (v))=c=p_i\,{\circ }\, \mu (\phi (u),\phi (v)), \end{aligned}$$

for each $i=1,2,\dots ,m$. This implies $\phi (u)$ and $\phi (v)$ are connected by an edge in $\varGamma _2$. So, $d_{\varGamma ^*_1}(u)=d_{\varGamma ^*_2}(\phi (u))$.

Now,

$$\begin{aligned} p_i\,{\circ }\, d_{\varGamma _1}(v)&=\sum _{u\ne v}p_i\,{\circ }\, \mu (u,v)\\&=\sum _{u\ne v}c\\&=cd_{\varGamma ^*_1}(v)\\&=cd_{\varGamma ^*_2}(\phi (v))\\&=p_iv d_{\varGamma _2}(\phi (v)), \text { for each } i=1,2,\dots ,m. \end{aligned}$$

This shows that the degree set of $\varGamma _1$ is equal to the degree set of $\varGamma _2$. Also, by the distance preserving property,

$$\begin{aligned} p_i\,{\circ }\, e_{\varGamma _1}(v)&=\vee \{p_i\,{\circ }\, \delta _1(u,v) |u\in {\tilde{A}}_1\}\\&=\vee \{p_i\,{\circ }\, \delta _2(\phi (u),\phi (v)) |u\in {\tilde{A}}_1\}\\&=p_i\,{\circ }\, e_{\varGamma _2}(\phi (v)), \text { for each } i=1,2,\dots ,m. \end{aligned}$$

This implies that the eccentricity set of $\varGamma _1$ is equal to the eccentricity set of $\varGamma _2$. Hence, $\varGamma _1$ and $\varGamma _2$ have the same degree and eccentricity set.

### Definition 13

Let $\varGamma =({\tilde{A}},\sigma ,\mu )$ be a *m*PFG. A *m*-polar fuzzy subgraph *H* of $\varGamma$ is isometric if $p_i\,{\circ }\, \delta _H(u,v)=p_i\,{\circ }\, \delta _\varGamma (u,v),$ for each $i=1,2,\dots ,m$ and for all $u, v\in H$.

### Definition 14

A *m*PFG $\varGamma =({\tilde{A}},\sigma ,\mu )$ is distance preserving if it has an isometric *m*-polar fuzzy subgraph with each possible number of vertices up to $|{\tilde{A}}|$.

### Theorem 6

If $\varGamma =({\tilde{A}},\sigma ,\mu )$ is a *m*PFG on a tree, then $\varGamma$ is a distance preserving *m*PFG.

### Proof

Let $\varGamma =({\tilde{A}},\sigma ,\mu )$ be a *m*PFG on a tree with *n* nodes. In a tree, a unique path exists between any two nodes.

If $\varGamma _1=({\tilde{A}}_1,\sigma _1,\mu _1)$ is a *m*-polar fuzzy subgraph obtained from $\varGamma$ by removing a vertex of degree 1 in $\varGamma ^*$, then using $\mu$-distance

$$\begin{aligned} p_i\,{\circ }\, \delta _{\varGamma _1}(u,v)=p_i\,{\circ }\, \delta _\varGamma (u,v), \end{aligned}$$

for each $i=1,2,\dots ,m$ and for all $u, v\in {\tilde{A}}_1.$

If $\varGamma _2=({\tilde{A}}_2,\sigma _2,\mu _2)$ is a *m*-polar fuzzy subgraph obtained from $\varGamma _1$ by removing a vertex of degree 1 in $\varGamma ^*_1$, then

$$\begin{aligned} p_i\,{\circ }\, \delta _{\varGamma _2}(u,v)=p_i\,{\circ }\, \delta _{\varGamma _1}(u,v), \end{aligned}$$

for each $i=1,2,\dots ,m$ and for all $u, v\in {\tilde{A}}_1$ and so on. This procedure gives an isometric *m*-polar fuzzy subgraph $\varGamma _j$ of $\varGamma$ with $|{\tilde{A}}|-j$, number of vertices, where $j=1,2,\ldots ,|{\tilde{A}}|-1$. Hence, $\varGamma$ is a distance preserving *m*PFG.

### Theorem 7

Isometry relation on *m*PFGs is a metric on a set of vertices.

### Proof

Let $\varGamma _1=({\tilde{A}}_1,\sigma _1,\mu _1)$ and $\varGamma _2=({\tilde{A}}_2,\sigma _2,\mu _2)$ be two *m*PFGs. Let $\varGamma _1$ be isometric to $\varGamma _2$. Then, each $v\in {\tilde{A}}_1$, there exists a bijection $f_v : {\tilde{A}}_1\rightarrow {\tilde{A}}_2$ such that $p_i\,{\circ }\,\delta _1(u,v)=p_i\,{\circ }\,\delta _2(f_v(u),f_v(v))$, for $i=1,2,\dots ,m$ and for all $u\in {\tilde{A}}_1$.

- Clearly, for each $v\in {\tilde{A}}_1$, $p_i\,{\circ }\,\delta _1(u,v)=p_i\,{\circ }\,\delta _2(f_v(u),f_v(v))\ge 0$, for each $i=1,2,\dots ,m$.
- Now, we take $u=v$, i.e., *u* and *v* have no path, then we have $p_i\,{\circ }\,\delta _1(u,v)=p_i\,{\circ }\,\delta _1(u,u)=p_i\,{\circ }\,\delta _2(f_v(u),f_v(u))=0$, for each $i=1,2,\dots ,m$. Again, $p_i\,{\circ }\,\delta _1(u,v)=0$ implies that *u* and *v* have no path i.e., $u=v$, for each $i=1,2,\dots ,m$.
- We know each $v\in {\tilde{A}}_1$, $p_i\,{\circ }\,\delta _1(u,v)=p_i\,{\circ }\,\delta _2(f_v(u),f_v(v))$, for $i=1,2,\dots ,m$ and for all $u\in {\tilde{A}}_1$. That is $p_1\,{\circ }\,\delta _2(f_v(u),f_v(v)), p_2\,{\circ }\,\delta _2(f_v(u),f_v(v)), \ldots , p_m\,{\circ }\,\delta _2(f_v(u),f_v(v))$, i.e., $p_1\,{\circ }\,\delta _1(u,v), p_2\,{\circ }\,\delta _1(u,v), \ldots , p_m\,{\circ }\,\delta _1(u,v)$, i.e., $p_1, p_2, \ldots , p_m$ be $u-v$ *m*-polar fuzzy paths, i.e., $p_1, p_2, \ldots , p_m$ be $v-u$ *m*-polar fuzzy paths, i.e., $p_1\,{\circ }\,\delta _1(v,u), p_2\,{\circ }\,\delta _1(v,u), \ldots , p_m\,{\circ }\,\delta _1(v,u)$, i.e., $p_1\,{\circ }\,\delta _2(f_v(v),f_v(u)), p_2\,{\circ }\,\delta _2(f_v(v),f_v(u)), \ldots , p_m\,{\circ }\,\delta _2(f_v(v),f_v(u))$, i.e., $p_i\,{\circ }\,\delta _2(f_v(v),f_v(u))=p_i\,{\circ }\,\delta _1(v,u)$, for each $i=1,2,\dots ,m$. So, $p_i\,{\circ }\,\delta _1(u,v)=p_i\,{\circ }\,\delta _1(v,u)$, for each $i=1,2,\dots ,m$.
- We know each $v\in {\tilde{A}}_1$, $p_i\,{\circ }\,\delta _1(u,v)=p_i\,{\circ }\,\delta _2(f_v(u),f_v(v))$, for $i=1,2,\dots ,m$ and for all $u\in {\tilde{A}}_1$. That is $p_1\,{\circ }\,\delta _2(f_v(u),f_v(v)), p_2\,{\circ }\,\delta _2(f_v(u),f_v(v)), \ldots , p_m\,{\circ }\,\delta _2(f_v(u),f_v(v))$, i.e., $p_1\,{\circ }\,\delta _1(u,v), p_2\,{\circ }\,\delta _1(u,v), \ldots , p_m\,{\circ }\,\delta _1(u,v)$, i.e., $p_1, p_2, \ldots , p_m$ be $u-v$ *m*-polar fuzzy path.

Again, for each $w\in {\tilde{A}}_1$, $p_i\,{\circ }\,\delta _1(v,w)=p_i\,{\circ }\,\delta _2(f_v(v),f_v(w))$, for $i=1,2,\dots ,m$ and for all $v\in {\tilde{A}}_1$. That is, $p_1\,{\circ }\,\delta _2(f_v(v),f_v(w)), p_2\,{\circ }\,\delta _2(f_v(v),f_v(w)), \ldots , p_m\,{\circ }\,\delta _2(f_v(v),f_v(w))$, i.e., $p_1\,{\circ }\,\delta _1(v,w), p_2\,{\circ }\,\delta _1(v,w), \ldots , p_m\,{\circ }\,\delta _1(v,w)$, i.e., $q_1, q_2, \ldots , q_m$ be $v-w$ *m*-polar fuzzy paths. Now, $p_1$ followed by $q_1$, $p_2$ followed by $q_2$, $\ldots$, $p_m$ followed by $q_m$ are $u-w$ *m*-polar fuzzy paths, each of which contains only one *m*-polar fuzzy path whose length cannot exceed $p_1\,{\circ }\,\delta _1(u,v)+p_1\,{\circ }\,\delta _1(v,w)$, $p_2\,{\circ }\,\delta _1(u,v)+p_2\,{\circ }\,\delta _1(v,w)$ and so on $p_m\,{\circ }\,\delta _1(u,v)+p_m\,{\circ }\,\delta _1(v,w)$ respectively. Thus we can write, $p_i\,{\circ }\,\delta _1(u,w)\le p_i\,{\circ }\,\delta _1(u,v)+p_i\,{\circ }\,\delta _1(v,w)$, for each $i=1,2,\dots ,m$.

## Antipodal concept on *m*-polar fuzzy graphs

In this section, we try to introduce a new idea of a *m*-polar fuzzy graph called antipodal *m*PFG. This is usually different from the concept of the *m*PFG. It mainly depends on $\mu$-distances and the diameter of a graph which is discussed in the previous section. Here, we also investigated many related properties about it.

### Definition 15

Let $\varGamma =({\tilde{A}},\sigma ,\mu )$ be a *m*PFG. Then an antipodal *m*PFG on *m*PFG $\varGamma$ is indicated as $A(\varGamma )=({\tilde{A}}_{A(\varGamma )},\sigma _{A(\varGamma )},\mu _{A(\varGamma )})$, where $p_i\,{\circ }\, \sigma _{A(\varGamma )}(u)=p_i\,{\circ }\, \sigma (u)$, for each $i=1,2,\dots ,m$. There exists an edge *u*, *v* in $A(\varGamma )$ if

$$\begin{aligned} p_i\,{\circ }\, \delta (u,v)=p_i\,{\circ }\, diam(\varGamma ) \end{aligned}$$

and $$p_i\,{\circ }\, \mu _{A(\varGamma )}(u,v) = \left\{ \begin{array}{llll} p_i\,{\circ }\, \mu (u,v),{when~ u,v~are~ neighbors~ in~} \varGamma \\ p_i\,{\circ }\, \sigma (u)\wedge p_i\,{\circ }\, \sigma (v), {when~ u,v~are~not~}\\ { neighbors~ in~} \varGamma .&{} \end{array}\right.$$ for all $i=1,2,\ldots ,m$.

Through the above formula we can calculate the edge MV.

**Note 1 **If $p_i\,{\circ }\, \delta (u,v)\ne p_i\,{\circ }\, diam(\varGamma )$, for all $u,v\in {\tilde{A}}$ and $i=1,2,\dots ,m$, then the corresponding antipodal *m*PFG $A(\varGamma )$ is called null graph.

### Definition 16

In antipodal *m*PFG, the degree of an edge is defined by $p_i\,{\circ }\, d_{A(\varGamma )}(u,v)=\sum _{(u,w)\in {\tilde{B}}_{A(\varGamma )}, u\ne w}p_i\,{\circ }\, \mu _{A(\varGamma )}(u,w)+ \sum _{(w,v)\in {\tilde{B}}_{A(\varGamma )}, w\ne v}p_i\,{\circ }\, \mu _{A(\varGamma )}(w,v)-2(p_i\,{\circ }\, \mu _{A(\varGamma )}(u,v)),$ for each $i=1,2,\dots ,m.$

### Definition 17

An antipodal *m*PFG $A(\varGamma )=({\tilde{A}}_{A(\varGamma )},\sigma _{A(\varGamma )},\mu _{A(\varGamma )})$ is edge regular if $p_i\,{\circ }\, d_{A(\varGamma )}(u,v)=k_i$ for each $i=1,2,\dots ,m$ and for all $(u,v)\in {\tilde{B}}$, i.e., every edge has same degree $(k_1,k_2,\ldots ,k_m)$, where $k_1,k_2,\ldots ,k_m$ are constants.

### Remark 1

If $\varGamma =({\tilde{A}},\sigma ,\mu )$ is an edge regular *m*PFG, then $A(\varGamma )=({\tilde{A}}_{A(\varGamma )}, \sigma _{A(\varGamma )},\mu _{A(\varGamma )})$ need not be an edge regular *m*PFG, which is explained below :

**Figure 2:**
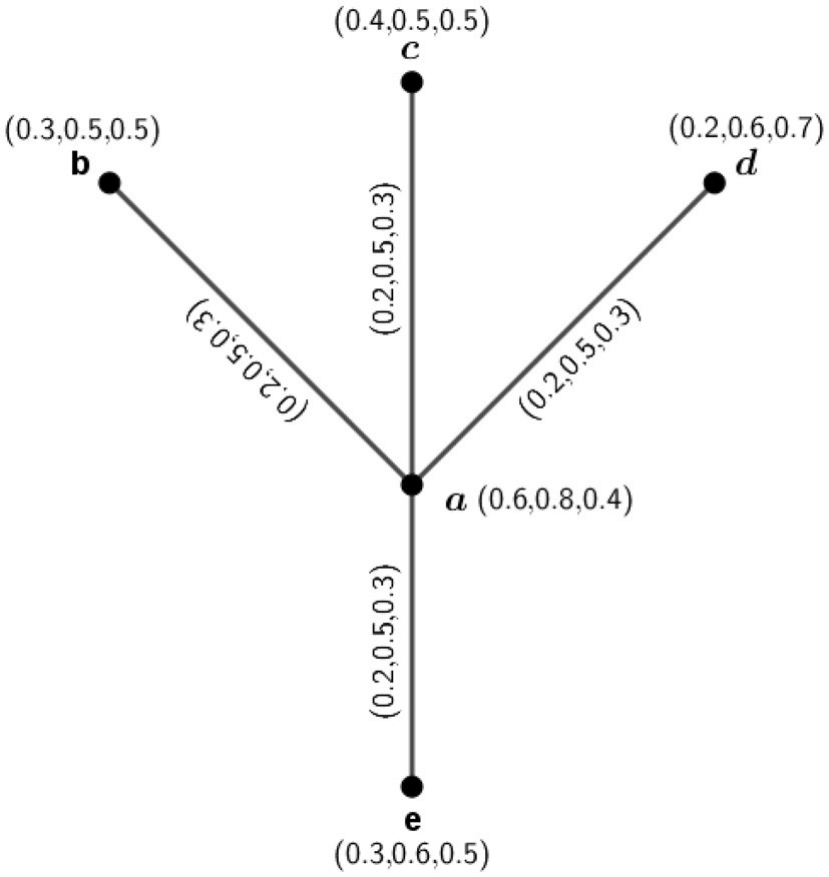
3PFG $$\varGamma _1=({\tilde{A}},\sigma ,\mu )$$.

### Example 3

Consider a 3PFG which is shown in Fig. 2. Here, $d_{\varGamma _1}(a,b)=4(0.2,0.5,0.3)+(0.2,0.5,0.3)-2(0.2,0.5,0.3)=(0.6,1.5,0.9)=d_{\varGamma _1}(a,c)=d_{\varGamma _1}(a,d)=d_{\varGamma _1}(a,e)$. Therefore, $\varGamma _1$ is an (0.6, 1.5, 0.9)-edge regular 3PFG. Now, the $\mu$-distances for the vertex *a* are $\delta (a,b)=(5,2,3.3),\delta (a,c)=(5,2,3.3),\delta (a,d)=(5,2,3.3)$ and $\delta (a,e)=(5,2,3.3)$. Therefore, the eccentricity of the node *a* is $e(a)=(5,2,3.3)$. Similarly, for vertex *b*, $\delta (b,a)=(5,2,3.3),\delta (b,c)=(10,4,6.6)=\delta (b,d)=\delta (b,e)$ and $e(b)=(10,4,6.6)$. For vertex *c*, $\delta (c,a)=(5,2,3.3),\delta (c,b)=(10,4,6.6)=\delta (c,d)=\delta (c,e)$. Therefore, $e(c)=(10,4,6.6)$. For vertex *d*, $\delta (d,a)=(5,2,3.3),\delta (d,b)=(10,4,6.6)=\delta (d,c)=\delta (d,e)$. Hence $e(d)=(10,4,6.6)$. For vertex *e*, $\delta (e,a)=(5,2,3.3),\delta (e,b)=(10,4,6.6)=\delta (e,c)=\delta (e,d)$, and $e(e)=(10,4,6.6)$. The diameter and radius of the graph are respectively $diam(\varGamma _1)=(10,4,6.6)$ and $r(\varGamma _1)=(5,2,3.3)$. Since, $diam(\varGamma _1)=(10,4,6.6)=\delta (b,c)=\delta (b,d)=\delta (b,e)=\delta (c,d)=\delta (c,e)=\delta (e,d)$, we can construct the antipodal *m*PFG for $\varGamma _1$.

**Figure 3:**
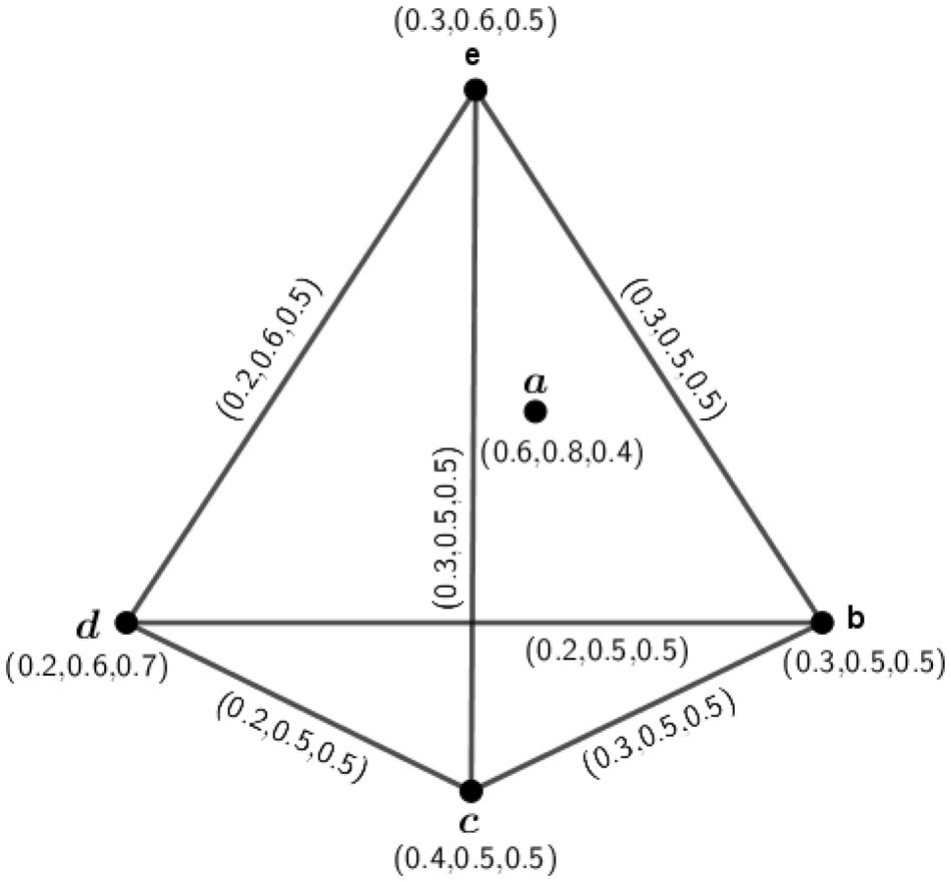
$A(\varGamma _1)=({\tilde{A}}_{A(\varGamma _1)},\sigma _{A(\varGamma _1)},\mu _{A(\varGamma _1)})$, for the graph of Fig. 2.

The MVs of vertices and edges of $A(\varGamma _1)$ are calculated below : for vertices, we know $\sigma _{A(\varGamma _1)}(a)=\sigma _{\varGamma _1}(a)$ for all $a\in {\tilde{A}}$, i.e., $\sigma _{A(\varGamma _1)}(a)=\sigma _{\varGamma _1}(a)=(0.6,0.8,0.4)$, $\sigma _{A(\varGamma _1)}(b)=(0.3,0.5,0.5)$, $\sigma _{A(\varGamma _1)}(c)=(0.4,0.5,0.5)$, $\sigma _{A(\varGamma _1)}(d)=(0.2,0.6,0.7)$ and $\sigma _{A(\varGamma _1)}(e)=(0.3,0.6,0.5)$. The edges of $A(\varGamma _1)$ are those pair of vertices *u* and *v*, $u,v\in {\tilde{A}}$, for which $\delta (u,v)=diam(\varGamma _1)$. Here, $diam(\varGamma _1)=\delta (b,c)=\delta (b,d)=\delta (b,e)=\delta (c,e)=\delta (d,e)$. Therefore the edge set of $A(\varGamma _1)$ is $\{(b,c),(b,d),(b,e),(c,e),(d,e)\}$. Now, by Definition 4.1, the MVs of the edges are $\mu _{A(\varGamma _1)}(b,c)=(0.3,0.5,0.5)$, $\mu _{A(\varGamma _1)}(b,d)=(0.2,0.5,0.5)$, $\mu _{A(\varGamma _1)}(b,e)=(0.3,0.5,0.5)$, $\mu _{A(\varGamma _1)}(c,d)=(0.2,0.5,0.5)$, $\mu _{A(\varGamma _1)}(c,e)=(0.3,0.5,0.5)$ and $\mu _{A(\varGamma _1)}(d,e)=(0.2,0.6,0.5)$. The graph $A(\varGamma _1)$ is shown in Fig. 3.

In $A(\varGamma _1)$, $d_{A(\varGamma _1)}(b,c)=(0.8,1.5,1.5)+(0.8,1.5,1.5)-2(0.3,0.5,0.5)=(1,2,2)$, $d_{A(\varGamma _1)}(e,c)=(1,2.1,2)$. So, $d_{A(\varGamma _1)}(b,c)\ne d_{A(\varGamma _1)}(e,c)$. Therefore, $A(\varGamma _1)$ is not an edge regular 3PFG.

### Remark 2

If $A(\varGamma )=({\tilde{A}}_{A(\varGamma )},\sigma _{A(\varGamma )},\mu _{A(\varGamma )})$ is an edge regular *m*PFG, then $\varGamma =({\tilde{A}},\sigma ,\mu )$ need not be an edge regular *m*PFG, which is justified in the following example.

**Figure 4:**
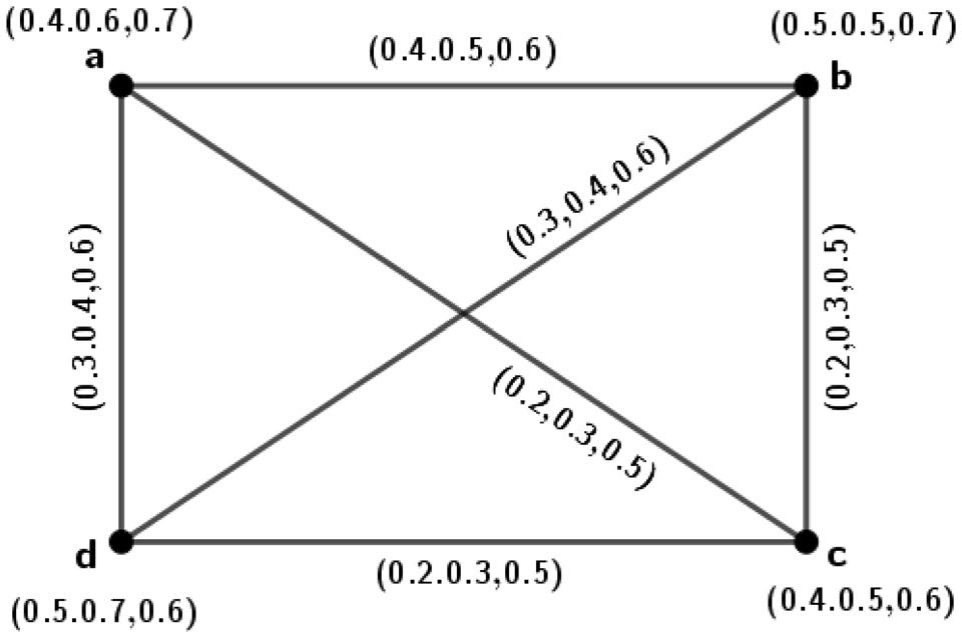
3PFG $$\varGamma _2=({\tilde{A}},\sigma ,\mu )$$.

### Example 4

Consider a 3PFG $\varGamma _2$, as shown in Fig. 4.

**Figure 5:**
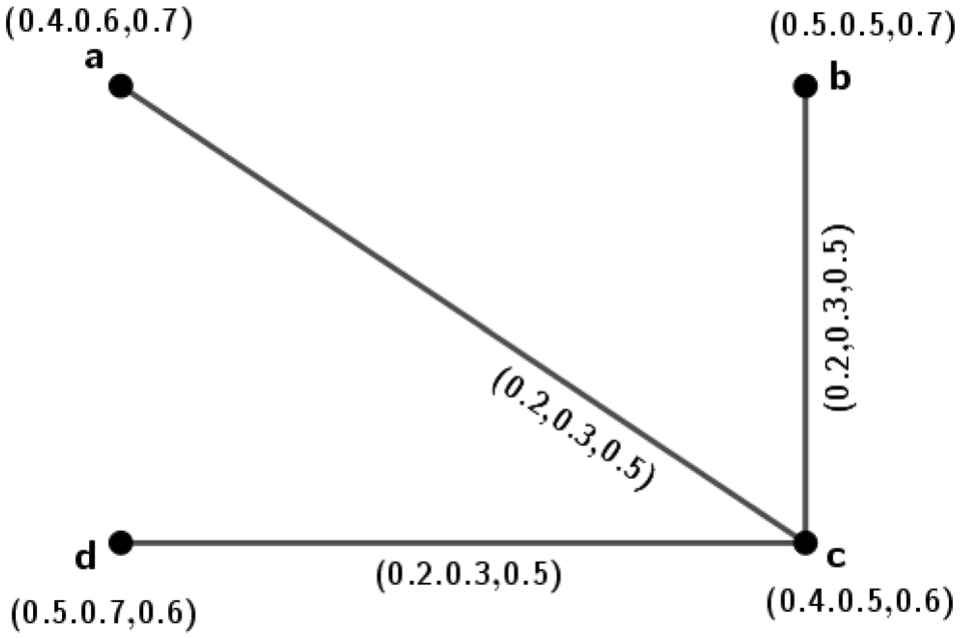
Antipodal 3PFG $$A(\varGamma _2)$$.

Here, $\delta (a,b)=(2.5,2,1.6), \delta (a,c)=(5,3.3,2), \delta (a,d)=(3.3,2.5,1.6), \delta (b,c)=(5,3.3,2), \delta (b,d)=(3.3,2.5,1.6)$ and $\delta (c,d)=(5,3.3,2)$. Therefore, $e(a)=(5,3.3,2)=e(b)=e(c)=e(d)$. Hence, $diam(\varGamma _2)=(5,3.3,2)$ and $r(\varGamma _2)=(5,3.3,2)$. Since, $diam(\varGamma _2)=\delta (a,c)=\delta (b,c)=\delta (c,d)$, by definition of antipodal *m*PFG, we can construct the graph $A(\varGamma _2)$ which is shown in Fig. 5.

In $A(\varGamma _2)$, $d_{A(\varGamma _2)}(d,c)=d_{A(\varGamma _2)}(a,c)=d_{A(\varGamma _2)}(b,c)=(0.4,0.6,1.0)$. So, $A(\varGamma _2)$ is an edge regular antipodal 3PFG but $\varGamma _2$ is not edge regular 3PFG because $d_{\varGamma _2}(a,b)=(1.0,1.4,2.2)\ne d_{\varGamma _2}(a,c)=(1.1,1.5,2.2)$.

### Theorem 8

Let $\varGamma =({\tilde{A}},\sigma ,\mu )$ be a complete *m*PFG such that $p_i\,{\circ }\, \mu$ is constant, for each $i=1,2,\dots ,m$. Then $A(\varGamma )$ is an edge regular *m*PFG.

### Proof

Since $\varGamma$ is complete and $p_i\,{\circ }\, \mu$ is constant, for each $i=1,2,\dots ,m$, $\varGamma$ is edge regular. Let $p_i\,{\circ }\, \mu (e)=c_i$, for each $i=1,2,\dots ,m$ and for all $e\in {\tilde{B}}$, where $c_1,c_2,\ldots ,c_m$ are constants. For any pair of distinct vertices $a,b\in {\tilde{A}}$, their $\mu$-distance $p_i\,{\circ }\, \delta (a,b)$ is $\frac{1}{c_i}$, for each $i=1,2,\dots ,m$. Then eccentricity of a vertex $a\in {\tilde{A}}$ is $p_i\,{\circ }\, e(a)=max_b(p_i\,{\circ }\, \delta (a,b))=\frac{1}{c_i}$, for each $i=1,2,\dots ,m$ and the diameter $p_i\,{\circ }\, diam(\varGamma )=max \{p_i\,{\circ }\, e(a)|a\in {\tilde{A}}\}=\frac{1}{c_i}$, for each $i=1,2,\dots ,m$. It follows that *a* and *b* are neighbors in $A(\varGamma )$, for all $a,b\in {\tilde{A}}$, which means that all the edges of $\varGamma$ are also a edge of $A(\varGamma )$. So by definition of antipodal *m*PFG, we have $\varGamma =A(\varGamma )$. Since $\varGamma$ is edge regular, $A(\varGamma )$ is an edge regular *m*PFG.

The converse of the above Theorem 8 need not be true, as explain in Example 5.

### Example 5

From Example 4, we see that $A(\varGamma _2)$ is edge regular but $p_i\,{\circ }\, \mu$, for each $i=1,2,\dots ,m$, is not a constant function. Therefore, the converse part need not be true.

### Remark 3

If $\varGamma =({\tilde{A}},\sigma ,\mu )$ is a complete *m*PFG, then $\varGamma$ need not be an edge regular *m*PFG, which is explained in the following example.

**Figure 6:**
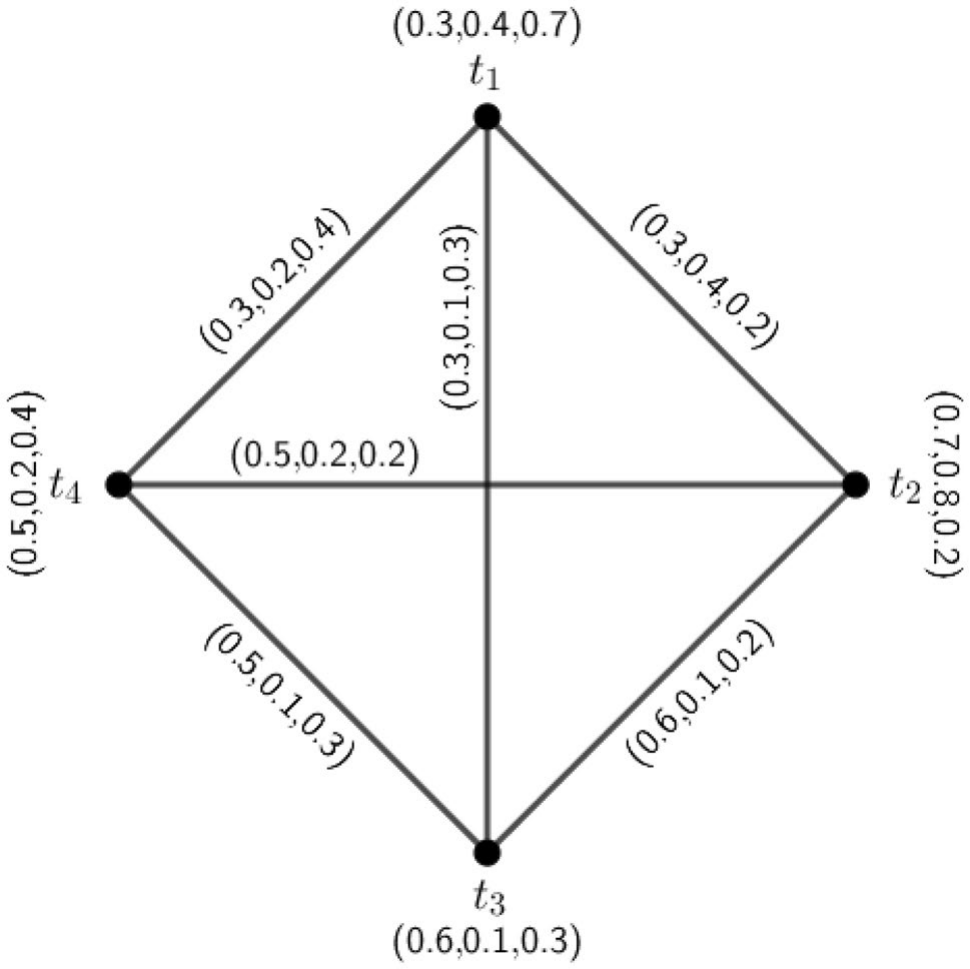
A 3PFG $\varGamma _3$ is complete but not edge-regular.

### Example 6

Consider a 3PFG $\varGamma _3$, as shown in Fig. 6.

From Fig. 6, clearly we see that the graph $\varGamma _3$ is complete. Now, the degree of edge $(t_1,t_2)=d(t_1,t_2)=(1.7,0.6,1.1)$ and the degree of edge $(t_1,t_3)=d(t_1,t_3)=(1.7,0.8,1.1)$. But $d(t_1,t_2)\ne d(t_1,t_3)$. This implies that $\varGamma _3$ is not an edge-regular *m*PFG.

### Remark 4

If $\varGamma =({\tilde{A}},\sigma ,\mu )$ is an edge regular *m*PFG with $p_i\,{\circ }\, \mu$ is a constant function, for each $i=1,2,\dots ,m$, then $A(\varGamma )$ need not be an edge regular *m*PFG, as discussed in the following example.

### Example 7

From Example 3, clearly, we see that $\varGamma$ is edge regular with $p_i\,{\circ }\, \mu$, for each $i=1,2,3$, is a constant function but $A(\varGamma )$ is not an edge regular 3PFG.

### Definition 18

Let $\varGamma =({\tilde{A}},\sigma ,\mu )$ be a *m*PFG and $A(\varGamma )$ be an antipodal *m*PFG of $\varGamma$. Now, the antipodal of $A(\varGamma )$ is denoted as $A(A(\varGamma ))$, simply, $A^2(\varGamma )$ and so on.

- If $\varGamma =A(\varGamma )$ then $\varGamma$ is called self-antipodal.
- If $A(\varGamma )=A^2(\varGamma )$ then $\varGamma$ is called idempotent in the sence of antipodal.
- If $A^n(\varGamma )=O$(null) for some positive integer *n*, then $\varGamma$ is called nilpotent in the sence of antipodal.

**Figure 7:**
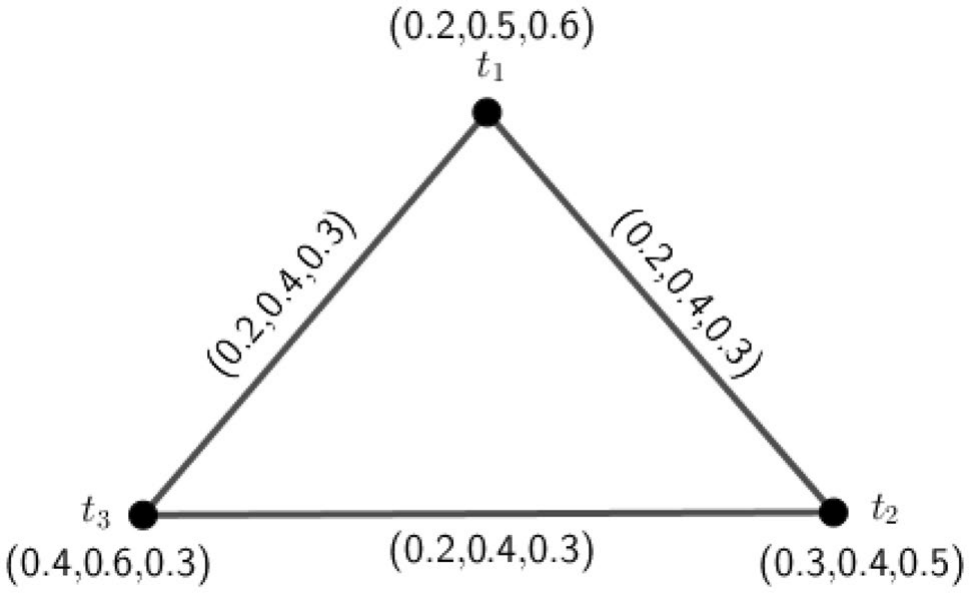
A 3PFG $\varGamma _4$ is a self-antipodal.

### Example 8

Consider a 3PFG $\varGamma _4$, as shown in Fig. 7.

Here, $\delta (t_1,t_2)=(5,2.5,3.3)$, $\delta (t_1,t_3)=(5,2.5,3.3)$, $\delta (t_2,t_3)=(5,2.5,3.3)$. Therefore $e(t_1)=(5,2.5,3.3)=e(t_2)=e(t_3)$. Hence, $diam(\varGamma _4)=(5,2.5,3.3)$. Since, $diam(\varGamma _4)=\delta (t_1,t_2)=\delta (t_1,t_3)=\delta (t_2,t_3)$, by definition of antipodal *m*PFG, we can say that $(t_1,t_2),(t_1,t_3)$ and $(t_2,t_3)$ are the edges of $A(\varGamma _4)$, whose MVs are same of $\varGamma _4$. We know all the vertices of $A(\varGamma _4)$ are same of $\varGamma _4$. Therefore, the antipodal graph $A(\varGamma _4)$ is same of $\varGamma _4$, i.e, $\varGamma _4=A(\varGamma _4)$. Hence, $\varGamma _4$ is a self-antipodal 3PFG.

Now, we have to show that $A^2(\varGamma _4)=A(\varGamma _4)$. Here, Fig. 7 is an antipodal 3PFG $A(\varGamma _4)$. In the similar way, we can say that all the vertices and edges of $A^2(\varGamma _4)$, antipodal of $A(\varGamma _4)$, are same of $A(\varGamma _4)$. So, $A(\varGamma _4)=A^2(\varGamma _4)$. Hence $\varGamma _4$ is an idempotent in the sence of antipodal.

**Figure 8:**
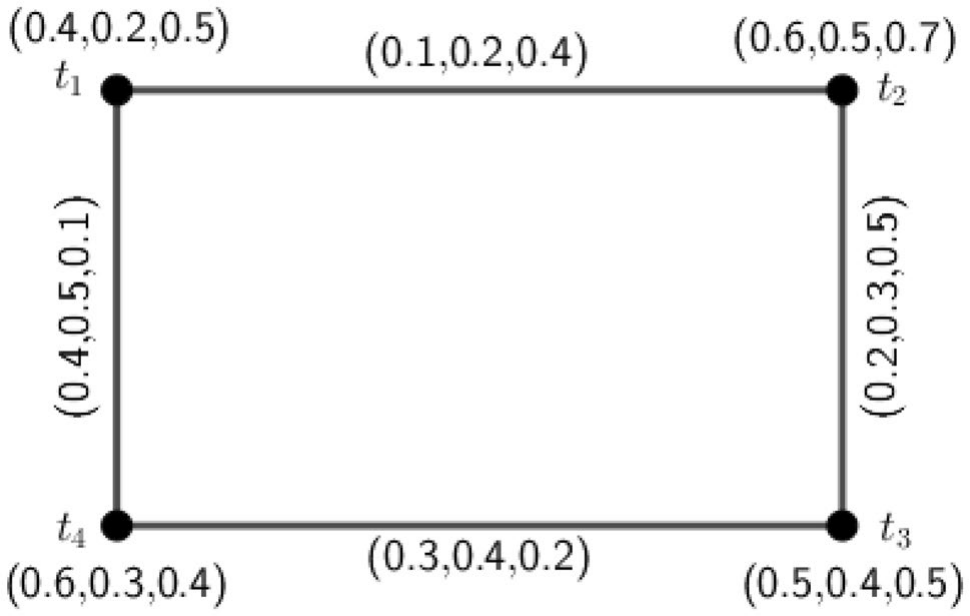
A 3PFG $\varGamma _5$ is nilpotent in the sence of antipodal.

### Example 9

Consider a 3PFG $\varGamma _5$, as shown in Fig. 8.

Here, $\delta (t_1,t_2)=(10,5,2.5)$, $\delta (t_1,t_3)=(5.8,4.5,4.5)$, $\delta (t_1,t_4)=(2.5,2,9.5)$, $\delta (t_2,t_3)=(5,3.3,2)$, $\delta (t_2,t_4)=(8.3,5.8,7)$, $\delta (t_3,t_4)=(3.3,2.5,5)$. Therefore $e(t_1)=(10,5,9.5),e(t_2)=(10,5.8,7),e(t_3)=(5.8,4.5,5)$ and $e(t_4)=(8.3,5.8,9.5)$. Hence, $diam(\varGamma _5)=(10,5.8,9.5)$. Since, $diam(\varGamma _5)\ne \delta (t_1,t_2)\ne \delta (t_1,t_3)\ne \delta (t_1,t_4)\ne \delta (t_2,t_3)\ne \delta (t_2,t_4)\ne \delta (t_3,t_4)$, by definition of antipodal *m*PFG, we can say that $(t_1,t_2),(t_1,t_3),(t_1,t_4),(t_2,t_3),(t_2,t_4)$ and $(t_3,t_4)$ are not the edges of $A(\varGamma _5)$. We know all the vertices of $A(\varGamma _5)$ are same of $\varGamma _5$. Therefore, the antipodal graph $A(\varGamma _5)$ contains only vertices. So this graph is a null graph, i.e, $A(\varGamma _5)=O$. Hence $A(\varGamma _5)$ is a nilpotent in the sence of antipodal.

### Theorem 9

Let $\varGamma =({\tilde{A}},\sigma ,\mu )$ be a complete *m*PFG such that $p_i\,{\circ }\, \mu$ is constant, for each $i=1,2,\dots ,m$. Then $A^n(\varGamma )$ is an edge regular *m*PFG, where *n* is a positive integer.

### Proof

Since $\varGamma$ is complete and $p_i\,{\circ }\, \mu$ is a constant function, for each $i=1,2,\dots ,m$, by Theorem 8, we have $\varGamma =A(\varGamma )=A^2(\varGamma )=A^3(\varGamma )=\ldots =A^n(\varGamma )$, for edge regularity. This implies that $A^n(\varGamma )$ is an edge regular *m*PFG, where *n* is a positive integer.

### Theorem 10

If $\varGamma =({\tilde{A}},\sigma ,\mu )$ is a complete *m*PFG such that $p_i\,{\circ }\, \sigma$ is a constant function, for each $i=1,2,\dots ,m$, then $A(\varGamma )$ is an edge regular *m*PFG.

### Proof

Since $\varGamma$ is complete and $p_i\,{\circ }\, \sigma$ is a constant function, for each $i=1,2,\dots ,m$, $p_i\,{\circ }\, \mu$ is also a constant function, for each $i=1,2,\dots ,m$. Hence the result follows from Theorem 8.

### Theorem 11

Let ${\tilde{C}}_{2n+1}=({\tilde{A}},\sigma ,\mu )$ be a *m*PFG on an odd cycle $C_{2n+1}=({\tilde{A}},{\tilde{B}})$ such that $p_i\,{\circ }\, \sigma$ is a constant function, for each $i=1,2,\dots ,m$. If ${\tilde{C}}_{2n+1}$ is an edge regular *m*PFG, then $A({\tilde{C}}_{2n+1})$ is an edge regular *m*PFG.

### Proof

Let ${\tilde{C}}_{2n+1}$ be an edge regular *m*PFG on an odd cycle $v_1v_2\dots v_{2n+1}v_1$. We know for an *m*PFG on an odd cycle $C_{2n+1}=({\tilde{A}},{\tilde{B}})$, ${\tilde{C}}_{2n+1}$ is edge regular if and only if $p_i\,{\circ }\, \mu$ is a constant function, for each $i=1,2,\dots ,m$. So $p_i\,{\circ }\, \mu$ is a constant function, for each $i=1,2,\dots ,m$. Let $p_i\,{\circ }\, \mu (e)=c_i$, for each $i=1,2,\dots ,m$ and for all $e\in {\tilde{B}}$, where $c_i$’s are constants. For any *j*, the distance from $v_j$ to the node farthest from $v_j$ in $C_{2n+1}$ is *n*. Since $p_i\,{\circ }\, \mu$ is a constant function, the $\mu$-distance from $v_j$ to the node farthest from $v_j$ in ${\tilde{C}}_{2n+1}$ is $n(\frac{1}{c_i})$, for each $i=1,2,\dots ,m$. Therefore, $p_i\,{\circ }\, e(v_j)=max_{v_k}(p_i\,{\circ }\, \delta (v_j,v_k))=n(\frac{1}{c_i})$, for each $i=1,2,\dots ,m$ and for all $v_j\in {\tilde{A}}$. Thus $p_i\,{\circ }\, diam({\tilde{C}}_{2n+1})=max\{p_i\,{\circ }\, e(v_j) |v_j\in {\tilde{A}}\}=\frac{n}{c_i}=p_i\,{\circ }\, e(v_j)$, for each $i=1,2,\dots ,m$ and for all $j=1,2,\dots ,2n+1$. Now, if $j\le n+1$, then the vertex farthest from $v_j$ is $v_{n+j}$. If $j> n+1$, then the vertex farthest $v_j$ is $v_{j-(n+1)}$. Hence $p_i\,{\circ }\, diam({\tilde{C}}_{2n+1})=p_i\,{\circ }\, e(v_j)$, where

$$\begin{aligned} p_i\,{\circ }\, e(v_j) = \left\{ \begin{array}{ll} p_i\,{\circ }\, \delta (v_j,v_{n+j}), &{}\text{ if } {} j\le n+1, \\ p_i\,{\circ }\, \delta (v_j,v_{j-(n+1)}), &{} \text{ if } {} j>n+1. \end{array}\right. \end{aligned}$$.

In $$A({\tilde{C}}_{2n+1})$$, $$v_1$$ is neighbor to $$v_{n+1}$$, $$v_2$$ is neighbor to $$v_{n+2}, \ldots , v_{n}$$ is neighbor to $$v_{2n}$$, $$v_{n+1}$$ is neighbor to $$v_{2n+1}$$, $$v_{n+2}$$ is neighbor to $$v_1$$, $$v_{n+3}$$ is neighbor to $$v_2$$, $$v_{n+4}$$ is neighbor to $$v_3$$, $$\dots$$ , $$v_{2n}$$ is neighbor to $$v_{n-1}$$, $$v_{2n+1}$$ is neighbor to $$v_{n}$$. Therefore, $$A({\tilde{C}}_{2n+1})$$ is an *m*PFG on an odd cycle $$v_1v_{n+1}v_{2n+1}v_{n}v_{2n}v_{n-1}\ldots v_{n+3}v_2v_{n+2}v_1$$. None of the edges in $$A({\tilde{C}}_{2n+1})$$ is in $${\tilde{C}}_{2n+1}$$. Let $$p_i\,{\circ }\, \sigma (v_j)=d_i$$, for each $$i=1,2,\dots ,m$$ and $$v_j\in {\tilde{A}}$$, where $$d_i$$’s are constants. Then $$p_i\,{\circ }\, \mu _{A({\tilde{C}}_{2n+1})}(u,v)=p_i\,{\circ }\, \sigma (u)\wedge p_i\,{\circ }\, \sigma (v)=d_i$$, for each $$i=1,2,\dots ,m$$ and for every edge (*u*, *v*) in $$A({\tilde{C}}_{2n+1})$$. We know for a *m*PFG $${\tilde{C}}_{2n+1}=({\tilde{A}},\sigma ,\mu )$$ on an odd cycle $$C_{2n+1}=({\tilde{A}},{\tilde{B}})$$, $${\tilde{C}}_{2n+1}$$ is edge regular if and only if $$p_i\,{\circ }\, 
\mu$$ is a constant function, for each $$i=1,2,\dots ,m$$. Hence $$A({\tilde{C}}_{2n+1})$$ is an edge regular *m*PFG.

The converse of the Theorem 11 need not be true, as shown in the following example.

### Example 10

**Figure 9:**
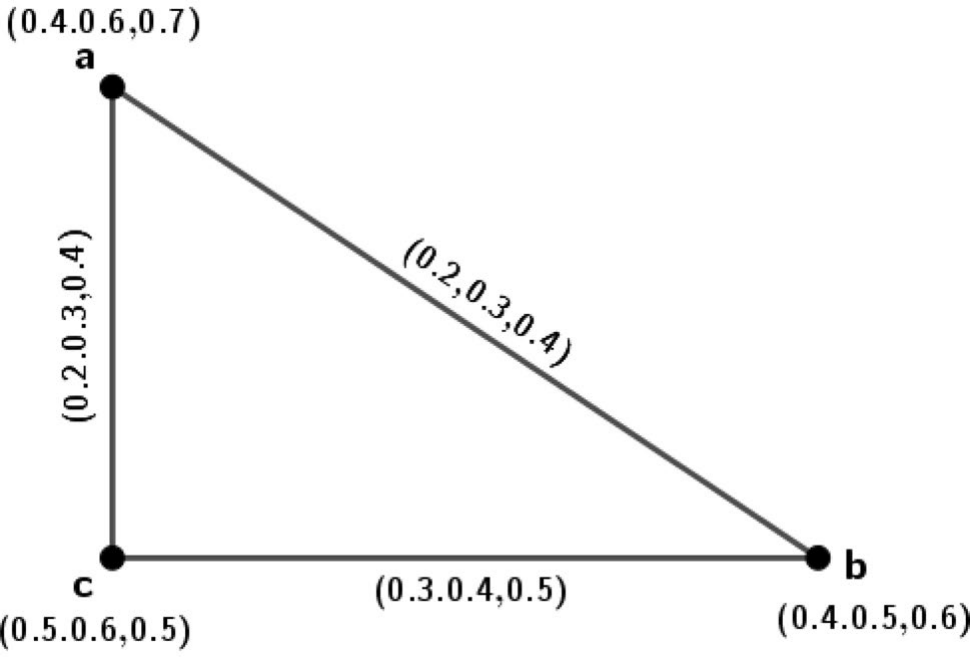
3PFG $$\varGamma =({\tilde{A}},\sigma ,\mu )$$.

Let us consider a 3PFG $\varGamma$, as shown in Fig. 9.

**Figure 10:**
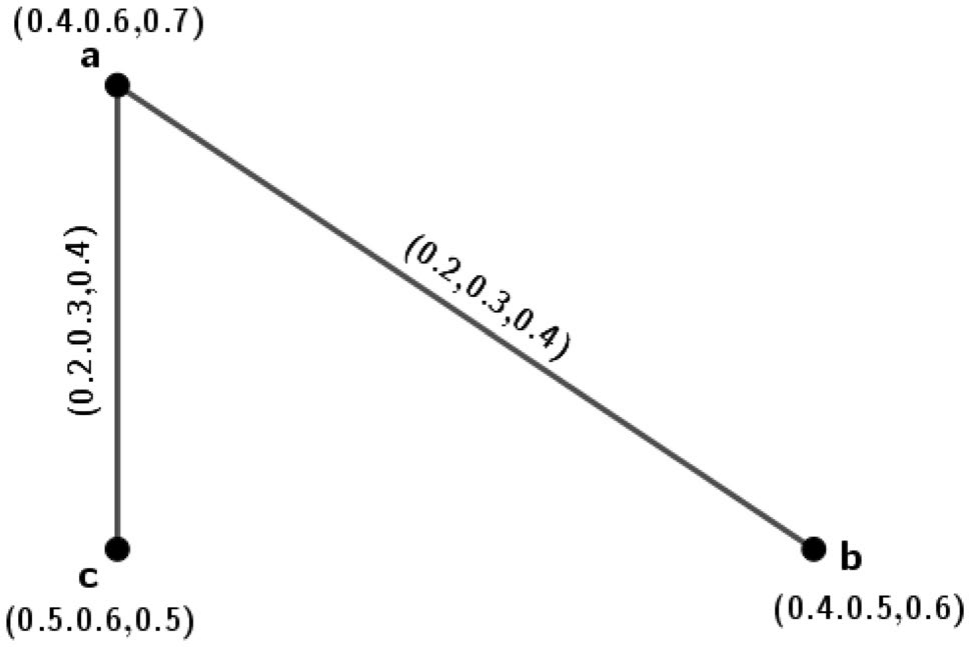
Antipodal 3PFG $$A(\varGamma )$$.

In $\varGamma$, the $\mu$-distances are $\delta (a,b)=(5,3.3,2.5)$, $\delta (a,b)=(5,3.3,2.5)$, $\delta (a,b)=(5,3.3,2.5)$. Now the eccentricity are $e(a)=(5,3.3,2.5),e(b)=(5,3.3,2.5)=e(c)$. So, $diam(\varGamma )=(5,3.3,2.5)$. Since, $diam(\varGamma )=\delta (a,b)=\delta (a,c)$, by definition of antipodal *m*PFG, we have drawn the graph $A(\varGamma )$ which is shown in Fig. 10.

Clearly, $A(\varGamma )$ is (0, 0, 0)-regular 3PFG when $\varGamma$ is not an edge regular 3PFG.

### Theorem 12

Suppose ${\tilde{C}}_{2n}=({\tilde{A}},\sigma ,\mu )$ be a *m*PFG on an even cycle $C_{2n}=({\tilde{A}},{\tilde{B}})$ with $n\not \equiv 0\pmod {2}$, where $|{\tilde{A}}|=2n$. If ${\tilde{C}}_{2n}$ is an edge regular *m*PFG, then $A({\tilde{C}}_{2n})=({\tilde{A}}_{A({\tilde{C}}_{2n})},\sigma _{A({\tilde{C}}_{2n})},\mu _{A({\tilde{C}}_{2n})})$ is a $(0,0,\ldots ,0)$-edge regular *m*PFG.

### Proof

Let ${\tilde{C}}_{2n}$ be an edge regular *m*PFG on an even cycle $v_1v_2\dots v_{2n}v_1$, with $n \not \equiv 0 \pmod {2}$. We know for an *m*PFG ${\tilde{C}}_{2n}=({\tilde{A}},\sigma ,\mu )$ on an even cycle $C_{2n}=({\tilde{A}},{\tilde{B}})$ with 2*n* vertices and $n \not \equiv 0 \pmod {2}$, ${\tilde{C}}_{2n}$ is an edge regular *m*PFG if and only if $p_i\,{\circ }\, \mu$ is a constant function, for each $i=1,2,\dots ,m$. So $p_i\,{\circ }\, \mu$ is a constant function, for each $i=1,2,\dots ,m$. Let $p_i\,{\circ }\, \mu (e)=c_i$, for each $i=1,2,\dots ,m$ and for all $e\in {\tilde{B}}$, where $c_i$’s are constants. For any *j*, the distance from $v_j$ to the node farthest from $v_j$ in $C_{2n}$ is *n*. Since $p_i\,{\circ }\, \mu$ is a constant function, the $\mu$-distance from $v_j$ to the node farthest from $v_j$ in ${\tilde{C}}_{2n}$ is $(n)(\frac{1}{c_i})$, for each $i=1,2,\dots ,m$. Therefore, $p_i\,{\circ }\, e(v_j)=max_{v_k}(p_i\,{\circ }\, \delta (v_j,v_k))=\frac{n}{c_i}$, for each $i=1,2,\dots ,m$ and for all $j=1,2,\dots ,2n$. Thus $p_i\,{\circ }\, diam({\tilde{C}}_{2n})=max\{p_i\,{\circ }\, e(v_j) |v_j\in {\tilde{A}}\}=\frac{n}{c_i}=p_i\,{\circ }\, e(v_j)$, for each $i=1,2,\dots ,m$ and for all $j=1,2,\dots ,2n$. Now $p_i\,{\circ }\, diam({\tilde{C}}_{2n})=p_i\,{\circ }\, e(v_j)$, where

$$\begin{aligned} p_i\,{\circ }\, e(v_j) = \left\{ \begin{array}{rcl} p_i\,{\circ }\, \delta (v_j,v_{j+n}), &{}\text{ if } &{} j\le n, \\ p_i\,{\circ }\, \delta (v_j,v_{j-n}), &{} \text{ if } &{} j> n. \end{array}\right. \end{aligned}$$

Therefore, $v_1v_{1+n}$, $v_2v_{2+n}$, $\ldots$ , $v_{n}v_{2n}$ are the only edges in $A({\tilde{C}}_{2n})$. Therefore every pair of distinct edges are non-adjacent in $A({\tilde{C}}_{2n})$. Hence $A({\tilde{C}}_{2n})$ is a $(0,0,\ldots ,0)$-edge regular *m*PFG.

The converse of the above Theorem 12 need not be true, as seen in the below example.

### Example 11

**Figure 11:**
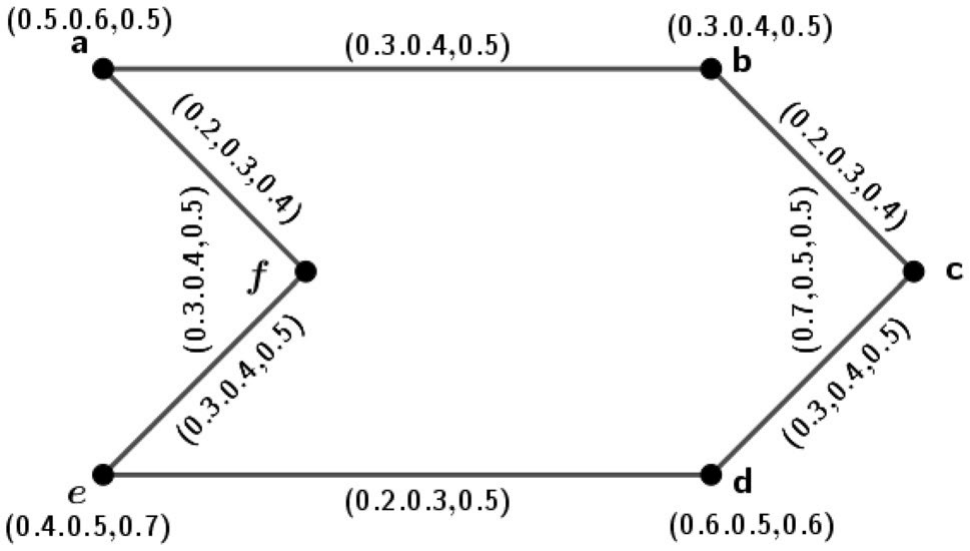
3PFG $$\varGamma =({\tilde{A}},\sigma ,\mu )$$.

**Figure 12:**
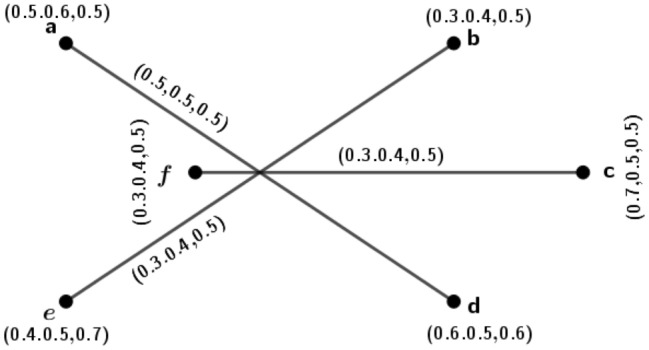
Antipodal 3PFG $$A(\varGamma )$$.

Consider a 3PFG $\varGamma$, as shown in Fig. 11, which is not an edge regular but the corresponding antipodal 3PFG $A(\varGamma )$ is a (0, 0, 0)-edge regular 3PFG, as shown in Fig. 12.

### Theorem 13

Let ${\tilde{C}}_{2n}=({\tilde{A}},\sigma ,\mu )$ be a *m*PFG on an even cycle $C_{2n}=({\tilde{A}},{\tilde{B}})$ with $2n \equiv 0 \pmod {4}$ and $n \equiv 0 \pmod {4}$, where $|{\tilde{A}}|=2n$. If ${\tilde{C}}_{2n}$ is an edge regular *m*PFG, then $A({\tilde{C}}_{2n})=({\tilde{A}}_{A({\tilde{C}}_{2n})},\sigma _{A({\tilde{C}}_{2n})},\mu _{A({\tilde{C}}_{2n})})$ is a $(0,0,\ldots ,0)$-edge regular *m*PFG.

### Proof

Let ${\tilde{C}}_{2n}$ be an edge regular *m*PFG on an even cycle $v_1v_2\dots v_{2n}v_1$, with $2n \equiv 0 \pmod {4}$ and $n \equiv 0 \pmod {4}$. We know for an *m*PFG ${\tilde{C}}_{2n}=({\tilde{A}},\sigma ,\mu )$ on an even cycle $C_{2n}=({\tilde{A}},{\tilde{B}})$ with 2*n* vertices and $2n \equiv 0 \pmod {4}$, then *G* is an $(k_1,k_2,\ldots ,k_m)$-edge regular *m*PFG if and only if $p_i\,{\circ }\, \mu$, for each $i=1,2,\dots ,m$, assumes exactly four values $r_i,s_i,t_i$ and $l_i$ such that consecutive adjacent edges receives these values in cyclic order with $r_i+t_i=k_i$ and $s_i+l_i=k_i$, for each $i=1,2,\dots ,m$. So, $p_i\,{\circ }\, \mu$ assumes exactly four values $r_i,s_i,t_i$ and $l_i$ in cyclic order and $n \equiv 0 \pmod {4}$, the $\mu$-distance from $v_j$ to the vertex farthest from $v_j$ in ${\tilde{C}}_{2n}$ is $(\frac{n}{2})(\frac{1}{r_i}+\frac{1}{s_i}+\frac{1}{t_i}+\frac{1}{l_i})$, for each $i=1,2,\dots ,m$. Thus $p_i\,{\circ }\, diam({\tilde{C}}_{2n})=\vee \{p_i\,{\circ }\, e(v_j) |v_j\in {\tilde{A}}\}=(\frac{n}{2})(\frac{1}{r_i}+\frac{1}{s_i}+\frac{1}{t_i}+\frac{1}{l_i})=p_i\,{\circ }\, e(v_j)$, for each $i=1,2,\dots ,m$ and for all $j=1,2,\dots ,2n$. Now $p_i\,{\circ }\, diam({\tilde{C}}_{2n})=p_i\,{\circ }\, e(v_j)$, where

$$\begin{aligned} p_i\,{\circ }\, e(v_j) = \left\{ \begin{array}{rcl} p_i\,{\circ }\, \delta (v_j,v_{j+n}), &{}\text{ if } &{} j\le n, \\ p_i\,{\circ }\, \delta (v_j,v_{j-n}), &{} \text{ if } &{} j> n. \end{array}\right. \end{aligned}$$

Therefore, $v_1v_{1+n}$, $v_2v_{2+n}$, $\ldots$ , $v_{n}v_{2n}$ are the only edges in $A({\tilde{C}}_{2n})$. Therefore every pair of distinct edges are non-adjacent in $A({\tilde{C}}_{2n})$. Hence $A({\tilde{C}}_{2n})$ is a $(0,0,\ldots ,0)$-edge regular *m*PFG.

## Real-life application of the proposed model

The *m*PFG is an important mathematical framework for presenting connected real-life phenomena through graphical models, where nodes and edges reside in *m*-polar fuzzy information. In this portion, by using the concept of $\mu$-length in *m*PFG, we have shown a road networking problem.

### Model construction

In the present scenario, graph theory has a vital role in solving many problems of network-based systems like social networks, road networks, gas pipeline networks and electric supply networks. For some models related to road networks, the optimal path problem is very significant. In road networking models, we mainly use the parameters travel distance as first component, travel time as second component and travel cost as third component in 3PFG model. Here, we assume a road network in which the crossings are indicated by nodes, and each edge indicates the road between two crossings. Here, we consider 8 nodes namely, $t_1,t_2,\ldots ,t_8$ to discus the proposed model.

The MVs of the vertices are considered based on the parameters like travel distance, travel time and travel cost coming from locality to crossing point. The MVs of all components of vertices are defined by the following way:

(i) First component of nodes is calculated through the following rules:- Between all the distances from locality to crossing points, let the maximum distance is *d* unit, say. This distances are taken as a first component of each crossing points in fuzzy form. Then it is calculated by the following way:
  $$\begin{aligned} \frac{\text {Actual~distance~between~locality~to~crossing~point}}{\text {Maximum~distance~from~locality~to~crossing~points}}\\ =\frac{\text {Actual~distance~between~locality~to~crossing~point}}{d}. \end{aligned}$$
(ii) For the second component, the maximum travel time from locality to crossing point is *t* unit, say. Then the MVs of second component are calculated as follows:
  $$\begin{aligned} \frac{\text {Actual~time~between~locality~to~crossing~point}}{\text {Maximum~time~from~locality~to~crossing~points}}\\=\frac{\text {Actual~time~between~locality~to~crossing~point}}{t}. \end{aligned}$$
(iii) For the third component,let the maximum traveling cost from locality to crossing point is *r* unit, say. Then the MVs of third component are calculated by the following rule:
  $$\begin{aligned} \frac{\text {Actual~travel~cost~between~locality~to~crossing~point}}{\text {Maximum~travel~cost~from~locality~to~crossing~points}}\\=\frac{\text {Actual~travel~cost~between~locality~to~crossing~point}}{r}. \end{aligned}$$
  So, the MVs of all components of each crossing point are given in Table 1.

**Table 1:** Vertex membership values of $\widetilde{\varGamma }$.

Vertex	Membership values
$t_1$	(0.4, 0.5, 0.3)
$t_2$	(0.5, 0.4, 0.2)
$t_3$	(0.3, 0.2, 0.1)
$t_4$	(0.4, 0.5, 0.3)
$t_5$	(0.7, 0.5, 0.2)
$t_6$	(0.3, 0.5, 0.1)
$t_7$	(0.6, 0.5, 0.4)
$t_8$	(0.7, 0.6, 0.2)

An *m*PFG is symmetric, i.e., $\mu {(t,u)}= \mu {(u,t)}$, where $\mu {(t,u)}$ represents the edge MV of the edge (*t*, *u*). ${\tilde{B}}=\{(t_1,t_2),(t_1,t_6),(t_1,t_8),(t_2,t_3),(t_3,t_4),(t_4,t_5),(t_4,t_7),(t_5,t_7),(t_5,t_6),(t_6,t_7),(t_3,t_7),(t_7,t_8),(t_2,t_8)\}$ is the 3PFS of roads. The MVs of the edges are considered depending on the parameters like travel distance, travel time and travel cost of journey. For illustration the MVs of edges are considered as in Table 2.

**Table 2:** Edge membership values of $\widetilde{\varGamma }$.

Edge	Membership values
$(t_1,t_2)$	(0.3, 0.4, 0.2)
$(t_1,t_6)$	(0.3, 0.3, 0.1)
$(t_1,t_8)$	(0.3, 0.3, 0.2)
$(t_2,t_3)$	(0.2, 0.2, 0.1)
$(t_2,t_8)$	(0.5, 0.4, 0.2)
$(t_3,t_4)$	(0.3, 0.2, 0.1)
$(t_3,t_7)$	(0.3, 0.1, 0.1)
$(t_4,t_5)$	(0.4, 0.5, 0.1)
$(t_4,t_7)$	(0.4, 0.4, 0.2)
$(t_5,t_6)$	(0.2, 0.2, 0.1)
$(t_5,t_7)$	(0.6, 0.4, 0.2)
$(t_6,t_7)$	(0.3, 0.4, 0.1)
$(t_7,t_8)$	(0.5, 0.4, 0.2)

**Figure 13:**
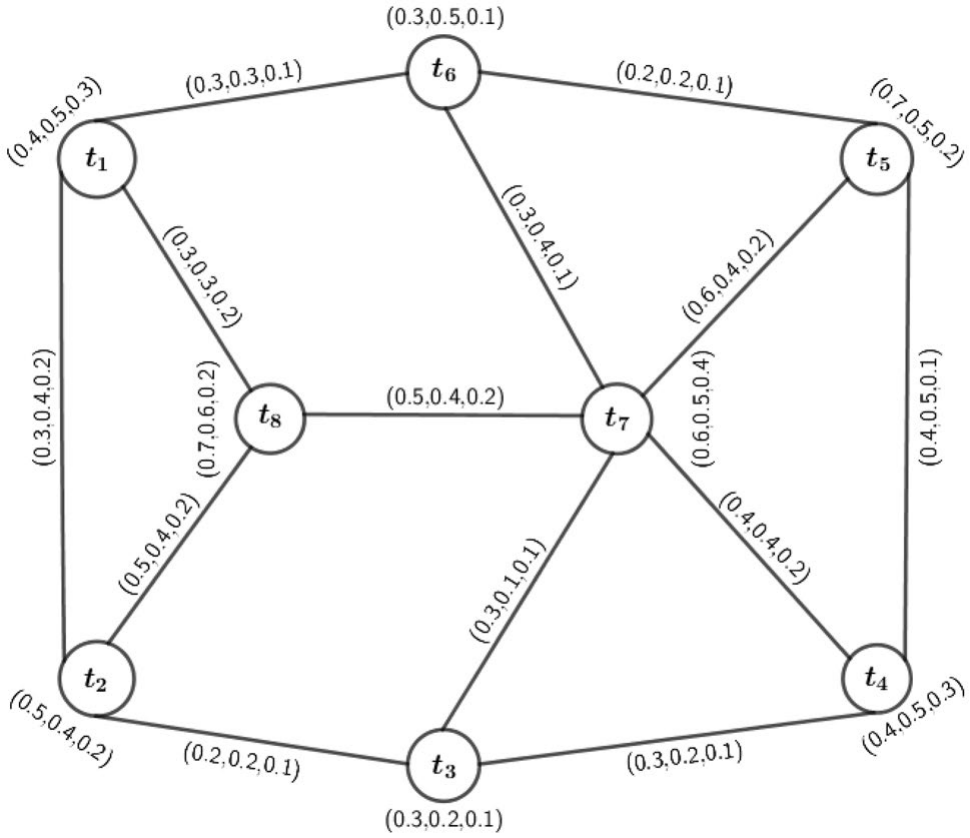
3-polar fuzzy model road network $\widetilde{\varGamma }$.

So the road network is based on vertices and edges depending on the parameters like travel distance, travel time and travel cost of journey are depicted by the model of 3PFG as seen in Fig. 13.

Based on the above parameters, we want to find out the suitable paths between the crossings.

### Determination of suitable paths

Here, we find out the suitable paths with respect to the parameters travel distance, travel time and travel cost between the crossings using the concept of $\mu$-length of *m*PFG. Now, we consider a path $\rho : t=t_0, t_1, t_2,\ldots , t_{n-1}, t_n=u$, for this the $\mu$-length is defined by

2 $$\begin{aligned} p_i\,{\circ }\, l(t,u)=\sum _{j=1}^{n}\frac{1}{p_i\,{\circ }\,\mu (t_{j-1},t_j)}, \end{aligned}$$

for each $i=1,2,\dots ,m$. So the $\mu$-distance, $p_i\,{\circ }\, \delta (t,u)$, is the smallest $\mu$-length of any $t-u$ path, for each $i=1,2,\dots ,m$. Generally, the normal length indicates the length of the road between two crossings. But, here the $\mu$-length between any two crossings indicates that distance which is taken based on the parameters. For this reason, we have considered this length for solving the problem. Now, from Fig. 13, it is easy to see that:

There are fourteen paths between $t_1$ and $t_3$ such that

$$\rho _1:t_1-t_2-t_3$$,

$$\rho _2:t_1-t_8-t_2-t_3$$,

$$\rho _3:t_1-t_8-t_7-t_3$$,

$$\rho _4:t_1-t_6-t_7-t_3$$,

$$\rho _5:t_1-t_6-t_7-t_8-t_2-t_3$$,

$$\rho _6:t_1-t_6-t_7-t_4-t_3$$,

$$\rho _7:t_1-t_6-t_7-t_5-t_4-t_3$$,

$$\rho _8:t_1-t_8-t_7-t_6-t_5-t_4-t_3$$,

$$\rho _9:t_1-t_8-t_7-t_5-t_4-t_3$$,

$$\rho _{10}:t_1-t_8-t_7-t_4-t_3$$,

$$\rho _{11}:t_1-t_6-t_5-t_7-t_8-t_2-t_3$$,

$$\rho _{12}:t_1-t_6-t_5-t_7-t_3$$,

$$\rho _{13}:t_1-t_6-t_5-t_4-t_7-t_3$$ and

$$\rho _{14}:t_1-t_6-t_5-t_4-t_3$$.

Now we evaluate the $\mu$-length of every path using Eq. (2). The $\mu$-length of $\rho _1$ is $l(\rho _1)=(p_1\,{\circ }\, l(\rho _1),p_2\,{\circ }\, l(\rho _1),p_3\,{\circ }\, l(\rho _1))=(\frac{1}{p_1\,{\circ }\,\mu (t_1,t_2)}+\frac{1}{p_1\,{\circ }\,\mu (t_2,t_3)},\frac{1}{p_2\,{\circ }\,\mu (t_1,t_2)}+\frac{1}{p_2\,{\circ }\,\mu (t_2,t_3)},\frac{1}{p_3\,{\circ }\,\mu (t_1,t_2)}+\frac{1}{p_3\,{\circ }\,\mu (t_2,t_3)})=(\frac{1}{0.3}+\frac{1}{0.2},\frac{1}{0.4}+\frac{1}{0.2},\frac{1}{0.2}+\frac{1}{0.1})=(8.3,7.5,15)$ and $\rho _2$ is $l(\rho _2)=(p_1\,{\circ }\, l(\rho _2),p_2\,{\circ }\, l(\rho _2),p_3\,{\circ }\, l(\rho _2))=(\frac{1}{0.3}+\frac{1}{0.5}+\frac{1}{0.2},\frac{1}{0.3}+\frac{1}{0.4}+\frac{1}{0.2},\frac{1}{0.2}+\frac{1}{0.2}+\frac{1}{0.1})=(10.3,10.6,20)$. Similarly, we can calculate the $\mu$-length of other paths which are $l\,(\rho _3)=(8.6,15.8,20)$, $l\,(\rho _4)=(9.9,15.8,30)$, $l\,(\rho _5)=(15.6,15.8,40)$, $l\,(\rho _6)=(12.4,13.3,35)$, $l\,(\rho _7)=(14,15.3,45)$, $l\,(\rho _8)=(19.4,20.3,50)$, $l\,(\rho _9)=(12.7,15.3,35)$, $l\,(\rho _{10})=(11.1,13.3,25)$, $l\,(\rho _{11})=(18.9,20.8,45)$, $l\,(\rho _{12})=(13.2,20.8,35)$, $l\,(\rho _{13})=(16.6,22.8,45)$ and $l\,(\rho _{14})=(14.1,15.3,40)$. The $\mu$-lengths of each path are listed in Table 3.

**Table 3:** $\mu$-lengths of the paths between $t_1$ and $t_3$.

Path	$\mu$-length
$$\rho _1$$	(8.3, 7.5, 15)
$\rho _2$	(10.3, 10.6, 20)
$\rho _3$	(8.6, 15.8, 20)
$\rho _4$	(9.9, 15.8, 30)
$\rho _5$	(15.6, 15.8, 40)
$\rho _6$	(12.4, 13.3, 35)
$$\rho _7$$	(14, 15.3, 45)
$\rho _8$	(19.4, 20.3, 50)
$\rho _9$	(12.7, 15.3, 35)
$\rho _{10}$	(11.1, 13.3, 25)
$\rho _{11}$	(18.9, 20.8, 45)
$\rho _{12}$	(13.2, 20.8, 35)
$\rho _{13}$	(16.6, 22.8, 45)
$\rho _{14}$	(14.1, 15.3, 40)

### Decision making for proposed model

Here, we discuss about suitable paths between the crossings $t_1$ and $t_3$ with respect to the parameters travel distance, travel time and travel cost of journey.

- Suitable path with respect to travel distance : From Table 3, we see that in path $\rho _1$, the distance is lowest, i.e, 8.3 unit. So, $\rho _1$ path is the most suitable path with respect to distance for journey. Similarly, the distance of the path $\rho _3$ is 8.6 unit, i.e, $\rho _3$ is the second most suitable path for journey.
- Suitable path with respect to travel time : From Table 3, we see that in path $\rho _1$, the travel time is minimum, i.e, 7.5 unit. So, $\rho _1$ path is the most suitable path with respect to travel time for journey. Similarly, the travel time of the path $\rho _2$ is 10.6 unit, i.e, $\rho _2$ is the second most suitable path for journey.
- Suitable path with respect to travel cost : From Table 3, we see that in path $\rho _1$, the traveling cost is minimum, i.e, 15 unit. So, $\rho _1$ is the most suitable path with respect to travel cost for traveling. Similarly, the paths $\rho _2$ and $\rho _3$, whose traveling cost is 20 unit, are the second most suitable paths with respect to travel cost for journey. Hence from the above discussion, $\rho _1:t_1-t_2-t_3$ is the most suitable path between two crossings $t_1$ and $t_3$ with respect to the parameters travel distance, travel time and travel cost for our journey. And second most suitable path with respect to distance is $\rho _3:t_1-t_8-t_7-t_3$, with respect to travel time is $\rho _2:t_1-t_8-t_2-t_3$, with respect to travel cost are $\rho _2$ and $\rho _3$. Hence, we can easily find out the suitable path between any two crossings by considering a 3PFG on a road network system.

## Advantages, disadvantages and limitations of the proposed work

In our real life, many problems have been solved using data which comes from different origins or sources. This type of data collection represents multi-polarity. In this type of polarity, we can not be structured well by the conception of fuzzy models, intuitionistic fuzzy models or bipolar fuzzy models. For example, if we consider a road networking model which assures minimum travel time of passengers. For this, we assign the node membership value (MV) based on the situation of the roads as (jam on the road, transport availability on the road, condition of the road). In nature, these terms are uncertain. To represent this situation, we need to use the 3-polar fuzzy model. Since, in our consideration, we consider three components for each nodes as well as edges, therefore we can not handle this type of situation using fuzzy model as their is a single components for this concept. Again, we can not apply bipolar or intuitionistic fuzzy graph model as each edges or nodes have just two components. Thus, these *m*PFG models give more efficient fuzziness results than other fuzzy model. Also, it is very interesting to develop and analyze such types of *m*PFGs with examples and related theorems. These definitions and theorems are definitely improving the existing concepts of *m*PFGs and are more reliable for solving any complicated real-life problem.

A few more advantages of the proposed model are:

(i) Anyone can analyze the membership values in a multi-polar fuzzy environment in this work in a certain way.
(ii) A real application of *m*-polar fuzzy model using the concept of $\mu$-length is presented on a road network system.
(iii) In isometric *m*PFG and antipodal *m*PFG environment, the conception of multi-polarity and the $\mu$-length together are studied.

The following are some of the disadvantages of the proposed:

(i) The negative MVs of the characters can not be used in this environment.
(ii) We can not use here non-heterogeneous types of data.

Some of the limitations of this study are given as follows:

(i) This work mainly focuses on isometry and antipodal concept in *m*-polar fuzzy environments.
(ii) If the MV of the characters are given in different interval-valued *m*-polar fuzzy environments, then the isometry and antipodal *m*-polar fuzzy graph cannot be used.
(iii) This type of proposed work mainly used in the networking system.

## Conclusion

In this paper, we discussed the *m*-polar fuzzy isometric graph along with many exciting facts about it. Metric space properties have also been implemented on *m*-polar fuzzy isometric graph. We also initiated a generalized fuzzy graph, namely antipodal *m*-polar fuzzy graphs, along with several facts. The degree of it is also presented along with edge regularity properties. We also give a relation between *m*-polar fuzzy antipodal graphs and their underlying crisp graphs. The properties have also been discussed on *m*-polar fuzzy odd as well as even cycles, complete graphs, etc. Finally, a real-life application based on a in *m*-polar fuzzy environment using the concept of $\mu$-length is also presented. In isometric *m*PFG and antipodal *m*PFG environment, the conception of multi-polarity and the $\mu$-length together are studied. Since, in our consideration, we consider three components for each nodes as well as edges, therefore we can not handle this type of situation using fuzzy model as their is a single components for this concept. Again, we can not apply bipolar or intuitionistic fuzzy graph model as each edges or nodes have just two components. Thus, these *m*PFG models give more efficient fuzziness results than other fuzzy model. Also, it is very interesting to develop and analyze such types of *m*PFGs with examples and related theorems. These definitions and theorems are definitely improving the existing concepts of *m*PFGs and are more reliable for solving any complicated real-life problem. The negative MVs of the characters can not be used in this environment. If the MV of the characters are given in different interval-valued *m*-polar fuzzy environments, then the isometry and antipodal *m*-polar fuzzy graph cannot be used. We will extend our future work depending on many other distances on the *m*-polar fuzzy environment. Also, we are interested in working with other types of distance problems mentioned in introduction part together with isometry and antipodal problem.

## Data Availability

The datasets used and/or analysed during the current study available from the corresponding author on reasonable request.

## Abbreviations

FG: Fuzzy graph
*m*PFG: *m*-polar fuzzy graph
UCG: Underlying crisp graph
MV: Membership value
*m*PFS: *m*-polar fuzzy set
